# SuperAging functional connectomics from resting-state functional MRI

**DOI:** 10.1093/braincomms/fcae205

**Published:** 2024-06-11

**Authors:** Bram R Diamond, Jaiashre Sridhar, Jessica Maier, Adam C Martersteck, Emily J Rogalski

**Affiliations:** Mesulam Center for Cognitive Neurology and Alzheimer’s Disease, Northwestern University Feinberg School of Medicine, Chicago, IL 60611, USA; Department of Psychiatry and Behavioral Sciences, Northwestern University Feinberg School of Medicine, Chicago, IL 60611, USA; Healthy Aging & Alzheimer’s Research Care (HAARC) Center, Department of Neurology, The University of Chicago, Chicago, IL 60637, USA; Mesulam Center for Cognitive Neurology and Alzheimer’s Disease, Northwestern University Feinberg School of Medicine, Chicago, IL 60611, USA; Department of Psychology, Florida State University, 1107 W Call St, Tallahassee, FL 32304, USA; Healthy Aging & Alzheimer’s Research Care (HAARC) Center, Department of Neurology, The University of Chicago, Chicago, IL 60637, USA; Healthy Aging & Alzheimer’s Research Care (HAARC) Center, Department of Neurology, The University of Chicago, Chicago, IL 60637, USA

**Keywords:** neuroimaging, successful aging, memory, neuropsychology

## Abstract

Understanding the relationship between functional connectivity (FC) of higher-order neurocognitive networks and age-related cognitive decline is a complex and evolving field of research. Decreases in FC have been associated with cognitive decline in persons with Alzheimer’s disease and related dementias (ADRD). However, the contributions of FC have been less straightforward in typical cognitive aging. Some investigations suggest relatively robust FC within neurocognitive networks differentiates unusually successful cognitive aging from average aging, while others do not. Methodologic limitations in data processing and varying definitions of ‘successful aging’ may have contributed to the inconsistent results to date. The current study seeks to address previous limitations by optimized MRI methods to examine FC in the well-established SuperAging phenotype, defined by age and cognitive performance as individuals 80 and older with episodic memory performance equal to or better than 50-to-60-year-olds. Within- and between-network FC of large-scale neurocognitive networks were compared between 24 SuperAgers and 16 cognitively average older-aged control (OACs) with stable cognitive profiles using resting-state functional MRI (rs-fMRI) from a single visit. Group classification was determined based on measures of episodic memory, executive functioning, verbal fluency and picture naming. Inclusion criteria required stable cognitive status across two visits. First, we investigated the FC within and between seven resting-state networks from a common atlas parcellation. A separate index of network segregation was also compared between groups. Second, we investigated the FC between six subcomponents of the default mode network (DMN), the neurocognitive network commonly associated with memory performance and disrupted in persons with ADRD. For each analysis, FCs were compared across groups using two-sample independent *t*-tests and corrected for multiple comparisons. There were no significant between-group differences in demographic characteristics including age, sex and education. At the group-level, within-network FC, between-network FC, and segregation measurements of seven large-scale networks, including subcomponents of the DMN, were not a primary differentiator between cognitively average aging and SuperAging phenotypes. Thus, FC within or between large-scale networks does not appear to be a primary driver of the exceptional memory performance observed in SuperAgers. These results have relevance for differentiating the role of FC changes associated with cognitive aging from those associated with ADRD.

## Teaser

Diamond *et al*. used resting-state functional MRI to compare SuperAgers (aged 80+ with superior episodic memory) to older controls with normal cognition and found no significant differences in functional connectivity within or between seven neurocognitive networks.

## Graphical Abstract

**Graphical Abstract:**
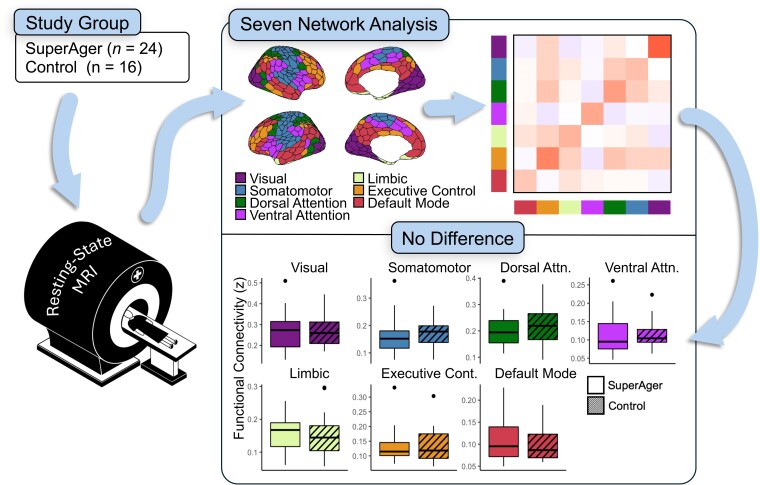

## Introduction

The global population is rapidly aging and the proportion of adults aged 85 or older is growing faster than younger generations.^1^ These adults are at the highest risk for both age-related memory decline^2^ and the onset of amnestic dementia due to Alzheimer’s disease (AD).^3^ However, significant memory decline is not inevitable. A growing literature on ‘successful aging’^4,5^ has aimed to describe and investigate older adults with unusually high physical,^6-8^ social,^7,9^ or cognitive functioning.^6,8^ Understanding mechanisms of successful cognitive aging promises to inform the development of interventions to prevent or slow cognitive decline.

One well-established phenotype of successful cognitive aging is *SuperAging,* defined by age and cognitive performance as individuals 80 years and older with episodic memory performance equal to or better than 50− to 60-year-olds.^10^ The multicenter SuperAging Research Initiative (R01AG045571, R01AG067781, and U19AG073153) was launched to understand what factors underlie this memory-specific phenotype and, by comparison, to inform our understanding of normal cognitive decline and AD. Initial results show that SuperAgers share unique neuropsychological,^11,12^ psychosocial,^13^ genetic,^10,14^ and biologic^15-19^ features. Post-mortem studies have identified neurobiological features of the SuperAger phenotype, such as relatively large neurons in the entorhinal cortex,^15^ a lower AD neuropathologic burden, and a greater density of von Economo neurons in the anterior cingulate cortex.^16^ Structural MRI studies have found brain features of the SuperAging phenotype that appear more like middle-aged-controls than older-aged-controls (OACs), such as relatively slow atrophy rates^20^ and thick cingulate cortices.^18^ To date, studies of the SuperAger phenotype have focused on brain morphometry. Less is known about the brain functional connectivity (FC) that supports their extraordinary memory abilities.

The brain is thought to be subdivided into distinct brain networks composed of highly interconnected neural regions that communicate to manifest complex behaviours and cognitive abilities.^21^ Resting-state functional MRI (rs-fMRI) has emerged as a proxy method for exploring the FC of these distributed neurocognitive networks^22^ by capturing intrinsic temporal correlations in neural activity^23^ indirectly measured from blood-oxygen-level-dependent (BOLD) signal. Exploratory rs-fMRI studies have identified resting-state networks that correspond to major networks previously established by neuroanatomical experiments^21,24^ and others with unclear neural connections.^25^ Some resting-state networks are thought to be involved in unimodal sensory processing, such as the visual^26^ and somatomotor networks.^23^ Other resting-state networks are thought to modulate indistinct higher-order cognitive functions, such as the frontoparietal,^27^ ventral attention,^28-30^ dorsal attention,^28^ and default mode networks (DMN).^31^

Resting-state networks have become a focus of cognitive aging research because they undergo a complex reorganization during development and in older adulthood^32,33^ and within-network FC has been directly related to cognitive performance.^29,34-36^ An age-related decrease in within-network FC has been demonstrated in regional activation during task-based fMRI comparing young and older adults^37,38^ and in cross-sectional studies of rs-fMRI over the normal lifespan as measured by independent component analysis^39^ and seed-based connectivity.^40-42^ The significance of these age-related changes is unclear. Some have suggested that a gradual shift away from ‘youthful’ functional patterns indicates progressive dysfunction and signifies or precipitates cognitive decline.^41,42^ One rationale for this hypothesis is that the topography of resting-state networks overlaps with regional activation during specific cognitive tasks. For example, commonly identified regions of the DMN, such as the inferior parietal lobe (IPL), posterior cingulate cortex (PCC), medial prefrontal cortex (MPC), parahippocampal cortex (PHC), medial temporal gyrus (MTG), reliably demonstrate greater BOLD activity during task-based fMRI involving episodic memory encoding and retrieval.^43-45^ The DMN is also commonly thought to include the hippocampus,^31^ the brain region most commonly associated with episodic memory.^46^ In some cases, within-network FC has also been related to composites of neurocognitive performances, such as persons with relatively strong within-network FC in the DMN performing better on episodic memory tasks.^34,39,41^ Nearly all higher-order resting-state networks display a similar pattern.^32^ As such, the overall degradation of within-network FC may be more closely related to the severe cognitive decline associated with ADRD.

The DMN has become a major focus of cognitive aging research because of its high detectability, topographical overlap with memory-related regions, and enigmatic association with AD dementia. The DMN is one of the most commonly studied resting-state networks.^47^ It demonstrates unique functional patterns in persons with mild neurocognitive impairments^48^ and amnestic dementia due to AD.^49^ The pathogenesis of AD amyloidopathy appears to selectively accumulate within regions of the DMN^50,51^ early in the disease course. Given these findings, some have argued that preserved DMN integrity in old age supports optimal memory abilities. However, DMN functional anomalies emerge prior to amnestic symptoms of AD.^49,52-55^ In addition, decreases in DMN cannot distinguish persons with amnestic AD from persons with non-amnestic variants of AD.^56-58^ Furthermore, functional changes in the DMN are also implicated in non-AD diagnoses including depression, autism spectrum disorder and schizophrenia^44^ where memory impairments are not core features. As such, the relationship between the DMN and memory decline in AD is complex, with evidence suggesting the network may be vulnerable to change in multiple disease-related states rather than being specific for AD.

Given the age and AD-related findings, it has recently been proposed that relatively robust FC within neurocognitive networks may support successful cognitive aging. Research differs in the terminology and classification of successful cognitive aging,^59,60^ specifically the age range of cohorts, which limits the generalizability of findings. Nonetheless, one recent study of adults aged 60 and over with exceptional episodic memory abilities found that these individuals had relatively strong FC within the DMN and ventral attention neurocognitive networks compared to similar aged cognitive controls.^34^ They also reported a positive association between FC and performance on a task of episodic memory. However, another recent study using the same methods in a separate but equally sized cohort from the Alzheimer’s Disease Neuroimaging Initiative (ADNI) database was unable to replicate their findings.^61^ Two recent studies have used machine learning to differentiate rs-fMRI signal from successful cognitive agers and controls in participants 60 and older^62^ and 80 and older.^63^ However, results are difficult to interpret because neither study has longitudinal data to ensure that participants were free from emergent neurodegenerative disease.

In summary, the role of resting-state networks in memory and aging is not well defined and the SuperAger phenotype provides a unique opportunity to understand its role in memory preservation beyond the eighth decade. The few studies that have investigated rs-fMRI in successful cognitive aging have had mixed results and no study has longitudinally monitored progressive cognitive decline. This study includes carefully characterized groups of cognitively stable SuperAgers and OACs over two or more visits to investigate the baseline functional integrity of seven canonically defined resting-state networks and subregions of the DMN.

## Materials and methods

### Participants

Retrospective data used in this project were obtained from the SuperAging Research Initiative database. The goal of the SuperAging Research Initiative is to identify factors that contribute to SuperAgers’ uniquely youthful memory function. Participants are community-dwelling, English speaking adults 80 years or older and without significant neurological or psychiatric illness. While enrolled, participants returned every two years for follow-up research visits. At each visit, participants receive a neuropsychological evaluation and, when feasible, MRI scans. Participants were recruited through community lectures, advertisements, word of mouth, community engagement and outreach activities, clinician referral and from the healthy control sample in the Clinical Core of the Alzheimer’s Disease Research Center (ADRC) at Northwestern University. This study was approved by the Institutional Review Boards of Northwestern University and the University of Chicago and informed consent was provided by all participants at enrolment.

### Neuropsychological evaluation

At each visit, we administered a battery of neuropsychological tests sensitive to detect cognitive aging and incipient amnestic AD dementia^64^ that capture both episodic memory and non-memory domains. Tests included the Rey Auditory Verbal Learning Test (RAVLT) for episodic memory, 30-item Boston Naming Test (BNT-30)^65^ for picture naming, Trail Making Test Part B (TMT-B)^66^ for executive functioning and Category Fluency Test^67^ for verbal fluency. Participant neuropsychological performance is summarized in Table 1.

**Table 1:** Participant demographic and cognitive characteristics

	SuperAgers	Older-aged controls	Statistic (*t*-test or $x^{2}$)	*P-*value
	(*n* = 24)	(*n* = 16)		
Demographic characteristics				
Age (SD), y	84.7 (2.89)	84.27 (3.67)	−0.4	0.69
Sex (F,M), no.	16, 8	10, 6	0.00	1.00
Handedness (R, L, A)	22, 1, 1	15, 1, 0	0.75	0.69^a^
Education (SD), y	16.79 (2.23)	15.88 (3.7)	−0.89	0.38
Follow-up time (SD), y	1.78 (0.31)	1.65 (0.3)	−1.31	0.2
Neuropsychological measures				
WTAR Est. FSIQ, (SD), SS	116.75 (6.24)	113.81 (8.83)	−1.15	0.26
RAVLT delay (SD), raw	11.25 (1.68)	6.13 (1.09)	−11.85	<0.001
CFT (SD), raw	22.29 (5.18)	18.25 (4.93)	−2.49	0.02
BNT-30 (SD), raw	28.71 (1.3)^b^	26.63 (3.36)	−2.36	0.03
TMT-B (SD), s	85.36 (33.48)	106.47 (53.01)^c^	1.38	0.18
MRI quality checking				
Mean FD (mm)	0.17 (0.05)	0.17 (0.04)	−0.12	0.91
Volumes after scrubbing (%)	94.31 (3.85)	93.55 (4.92)	−0.53	0.6

^a^One SuperAger was ambidextrous and not included in the $x^{2}$ analysis.

^b^One SuperAger met all SuperAging criteria with the exception of the BNT-30, but scored within expectation in a subsequent visit, 6 months later.

^c^One older-aged control was missing the TMT-B at the MRI visit.

SD = standard deviation; y = years; F = female; M = male; d = days; R = right; L = left; A = ambidextrous; SS = standard score (mean = 100; SD = 15); WTAR = Wechsler test of adult reading; FSIQ = Full-Scale Intelligence Quotient; RAVLT = Rey Auditory Verbal Learning Test; CFT = Category Fluency Test; TMT-B = Trail Making Test - B; BNT-30 = 30-item Boston Naming Test; FD = Framewise Displacement.

### Group criteria

In accordance with the criteria operationally defined for SuperAgers in the study by Harrison *et al*., 2012, SuperAgers performed above the average range for their peer age normative group on the RAVLT delayed recall (raw score, ≥ 9), at least as good as normative scores for adults in their 50s and 60s,^68^ and at least in the average range compared to same-aged peers on the three other tests loading on non-memory cognitive domains (BNT, TMT-B and Category Fluency Test). OAC scored within one standard deviation of average compared to same-aged peers on the memory measure (RAVLT delayed recall score between 3 and 7) and at least in the average range on other cognitive tests. Group classification criteria are summarized in Table 2. Participants maintained group status at consecutive study visits occurring approximately two years apart (mean = 1.7 years; range = 1.16–2.29 years).

**Table 2:** Group classification criteria according to neuropsychological test scores

Cognitive domain	Neuropsychological assessment	SuperAger	Older-aged control
Episodic memory	RAVLT	ss ≥ 10^a^	ss = 7–11
Executive Functioning	Trail Making Test: Part B	ss ≥ 7	ss ≥ 7
Verbal fluency	Semantic Fluency: Animals	T ≥ 40	T ≥ 40
Picture naming	BNT-30	ss ≥ 7	ss ≥ 7

^a^Scaled scores for SuperAgers are compared to 56–64-year-olds (midpoint age = 61); all other standardized scores are compared to same-aged peers.

Standardize score summary statistics: ss (mean = 10; SD = 3), *T* (mean = 50; SD = 10). Reference norms: Rey Auditory Verbal Learning Task, Trail Making Test: Part B, Semantic Fluency, Boston Naming Test. SD = standard deviation; ss = Scaled Score; T = T-Score.

### Inclusion criteria

**Figure 1:**
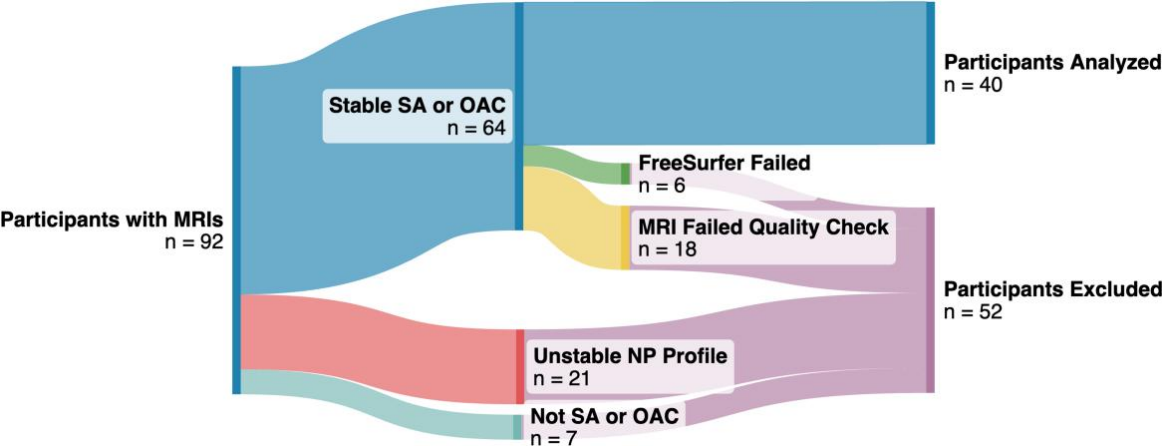
**Flow chart of cohort selection.** T1w = T1-weighted; rs-fMRI = resting-state functional magnetic resonance imaging; NP = neuropsychological; SA = SuperAger; OAC = older-aged control.

Participants with T_1_-weighted (T1w) scans and rs-fMRI scans were considered for this study. Of these participants, we identified those with stable neuropsychological profiles over two visits and excluded those whose group status changed over that interval of time (e.g. participants who developed mild cognitive impairment). MRIs were collected during both visits. Data from the baseline MRI were used in our analysis where possible; data from the second MRI was used only if the baseline scan was unavailable or unusable. We excluded participants with scans containing artefacts in both scans (e.g. magnetic susceptibility, motion, aliasing). In total, 40 participants [mean age (SD): 84.5 (3.2)] were identified for inclusion in the present study. Participant demographics are summarized in Table 1 and a flow chart detailing cohort selection is shown in Fig. 1.

### Imaging protocol

MRI scans were acquired for all participants with a standard 12-channel birdcage head coil on a Siemens 3T MAGNETOM TIM Trio scanner (Erlangen, Germany). For surface reconstruction, we acquired structural T1w MP-RAGE sequences (repetition time [TR] = 2300 ms, echo time [TE] = 2.86 ms and flip angle = 9°, 1 mm^3^). The functional scan was un-directed and participants were instructed to stay awake, keep their eyes open and let their minds wander. Runs were 11.5-minute long and consisted of a spin echo/echo planar imaging sequence with 244 volumes (TR = 2800 ms, TE = 20 ms, 1.7 × 1.7 × 3 mm^3^).

### MRI processing

#### Structural imaging

T1w images underwent volumetric segmentations and surface reconstruction by FreeSurfer (v7.2). Trained technicians visually inspected and made iterative edits to optimize FreeSurfer processing. Volumetric segmentations and surface reconstructions were used for registration during rs-fMRI processing.

#### fMRI quality assessment

Motion artefacts in rs-fMRI are concerning for confounds in older populations.^69,70^ Quality checking measures were used to censor time series motion artefacts by accounting for deviation in frame-to-frame motion and signal. Respectively, framewise displacement (FD) is a six-dimensional metric of instantaneous head motion calculated from frame-to-frame and DVARS is the relative change in signal from frame-to-frame. We used the eXtensible Connectivity Pipeline^71,72^ (XCP; v3.2) to calculate FD using the formula from Power *et al*. (2014), with a head radius of 50 mm. Volumes with filtered FD greater than 0.4 mm were flagged as outliers and excluded from nuisance regression. The filtered versions of the motion traces and FD were not used for denoising. DVARS and the correlation between DVARS and FD decreased following motion scrubbing. Scans with fewer than 80% interpretable frames (total scan-time, ≥ 9 minutes) were excluded. Quality reports produced by fMRIPrep^73^ (v22.1.1) and XCP^71,72^ were inspected to ensure suitable completion of preprocessing steps. Quality checking metrics are summarized in Table 1.

#### fMRI processing

Minimal functional MRI preprocessing was performed using fMRIPrep^73^ with custom methodologies. fMRIPrep preprocessing included slice-time correction, motion correction using affine registration to the middle time-point, co-registration to the T1w image and resampling into standard space using a single interpolation step. Following minimal MRI preprocessing, XCP^71,72^ was used to post-process rs-fMRI. XCP post-processing includes the removal of initial rs-fMRI volumes, outlier detection and filtering, de-spiking and interpolation and a bandpass filter (0.01–0.1 Hz) to reduce low-frequency drift and high-frequency noise in the signal. Global signal, the first principal components from cerebral spinal fluid and white matter, and frame-to-frame motion in six degrees of freedom calculated during motion correction were regressed out to reduce physiologic noise.^74^ The use of global signal regression is controversial and may remove real neural signal.^75^ Therefore, all analyses were replicated using rs-fMRI without global signal regression. The processed BOLD was smoothed on the surface using Connectome Workbench^76^ with a Gaussian kernel size of 3.0 mm in agreement with best practices.^77-80^

**Figure 2:**
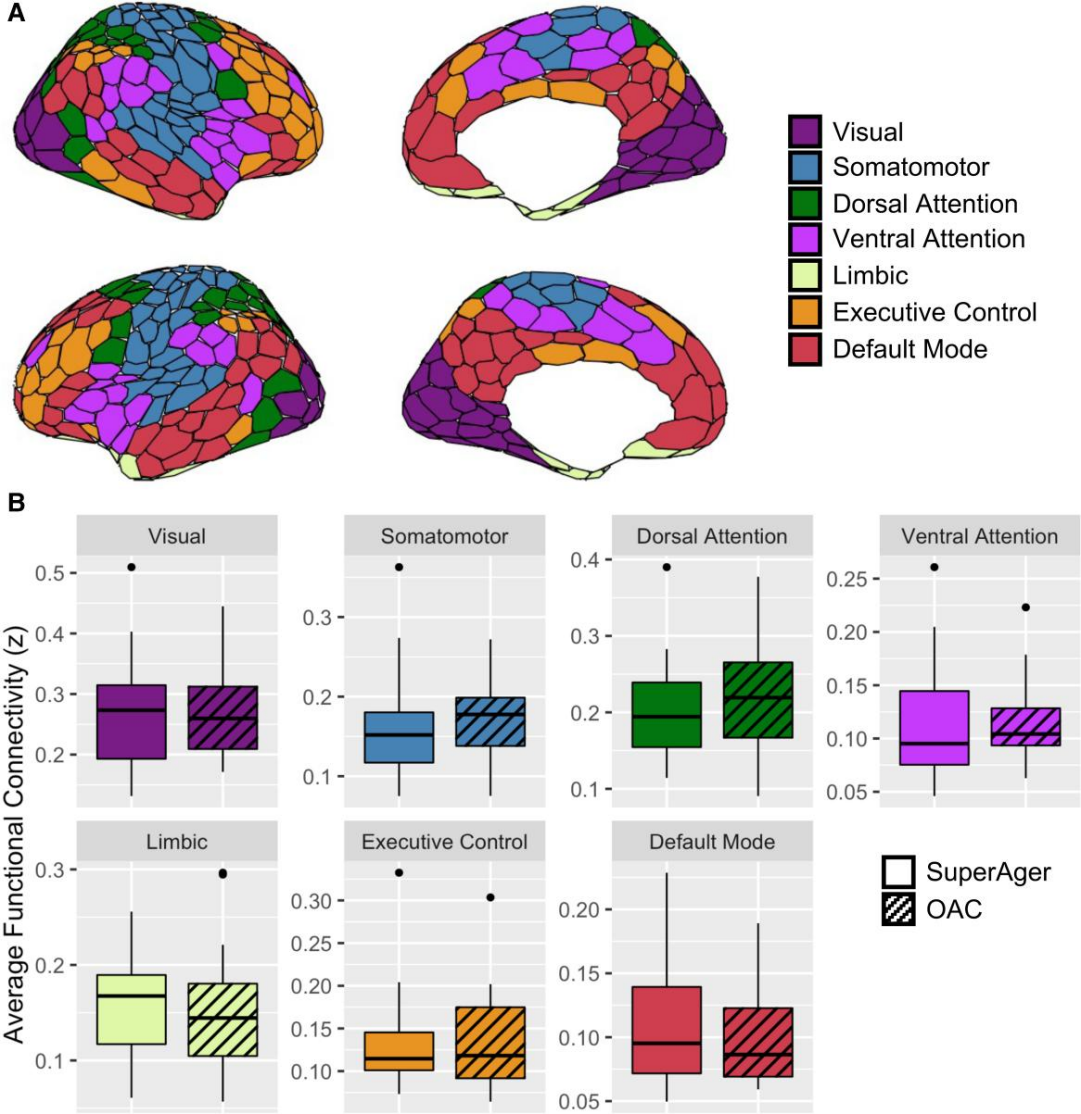
**Within-network resting-state functional connectivity does not differ between groups.** (**A**) 400-parcel cortical parcellation of seven large-scale resting-state networks from Schaefer *et al*. (2018) used for within-network analysis. (**B**) Average within-network functional connectivity across resting-state networks did not differ between SuperAgers and older-aged controls; OAC = older-aged controls. Wilcoxon signed-rank test; *P* > 0.05 for all comparisons.

To calculate within-network FC of the DMN, executive control, limbic, ventral attention, dorsal attention, somatomotor and visual networks, Connectome Workbench was used to extract residual signal from all parcels of a seven network (see Fig. 2A), 400 region-of-interest (ROI) parcellation (Schaefer *et al*., 2018), of a common group-level resting-state atlas (Yeo *et al*., 2011), resulting in 400 time-series.^25,81^ Subsequently, pair-wise FC (i.e. Pearson’s *r* converted to Fisher’s *z*) between all ROI time-series were computed to create connectivity matrices (400 × 400). Within-network FC was defined as the average *z*-value for all pairs within a given network. The seven network parcellation from Schaefer *et al*. (2018) is freely available online.^81^

**Figure 3:**
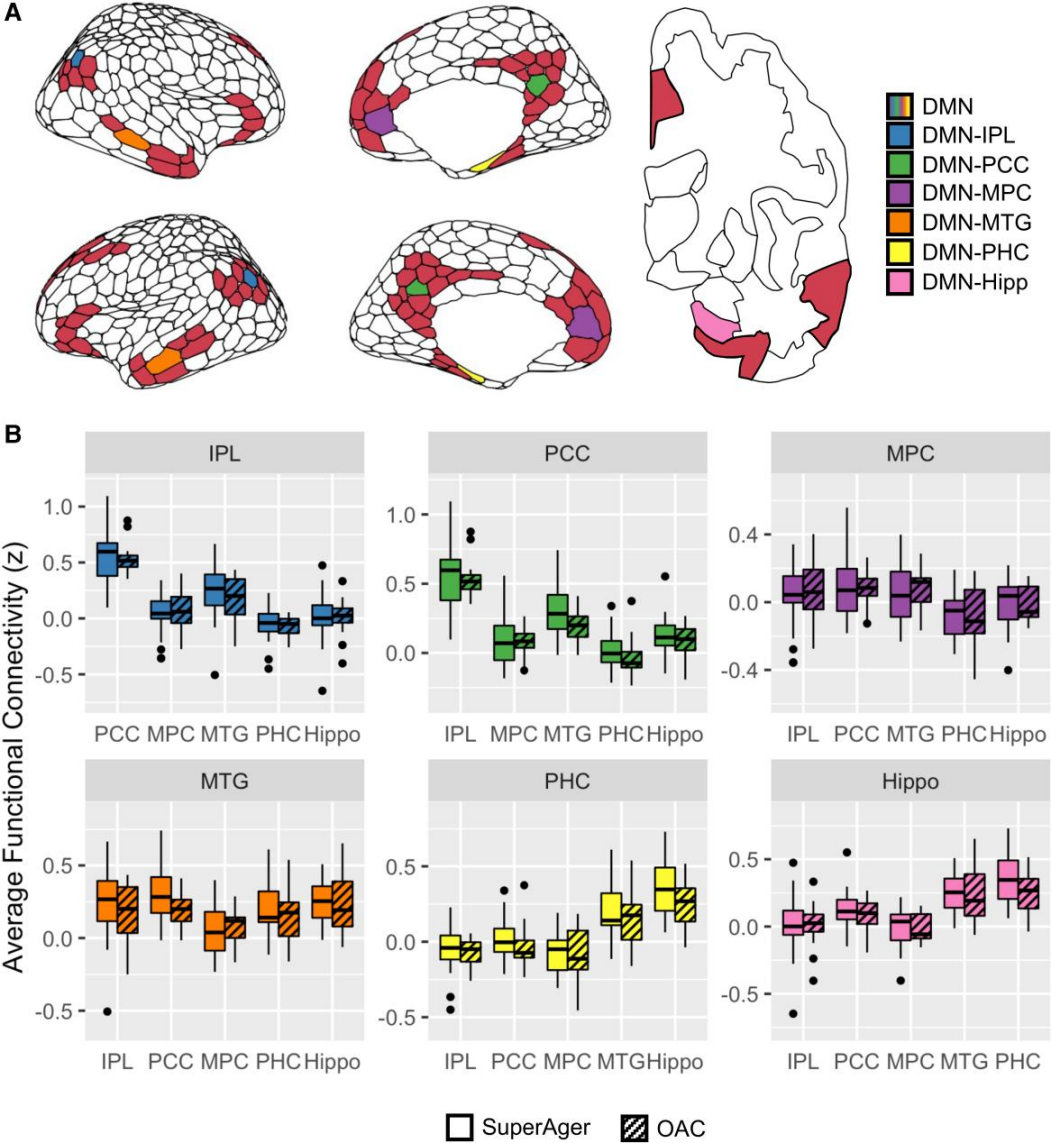
**Functional connectivity between subcomponents of the DMN do not differ between SuperAgers and controls.** (**A**) Regions-of-interest (ROIs) used for functional connectivity of the default mode network (DMN); Left: Five central polygons from DMN clusters of the 600-parcel cortical parcellation from Schaefer *et al*., 2018; Right: Hippocampus (Hipp) seed from Glasser *et al*., 2013. (**B**) Two-sample independent *t*-tests found no significant between-group difference in functional connectivity; IPL = inferior parietal lobe; PCC = posterior cingulate cortex; MPC = medial prefrontal cortex; PHC = parahippocampal cortex; MTG = middle temporal gyrus. Wilcoxon signed-rank test; *P* > 0.05 for all comparisons.

To calculate the DMN subcomponent FC, ROI-ROI correlation coefficients were calculated from six bilateral DMN canonical regions: the IPL, PCC, MPC, PHC, MTG and hippocampus. Correlations were calculated between each possible pair, for a total of 30 correlations (15 unique correlations) per person. To ensure that ROIs were completely confined to their predefined regions, cortical areas of interest were defined as the central polygon for each DMN region from the seventeen network parcellations subdivided into 600 parcels^81^ (Supplemental Fig. 1). The hippocampal ROI was taken from the Human Connectome Project (HCP) subcortical CIFTI atlas.^78^ All ROIs were chosen to include homologous regions on both hemispheres. ROI placement is shown in Fig. 3A and cortical ROI identification values (corresponding to the atlas CIFTI metadata) are provided in Supplemental Table 1. Code to generate the custom CIFTI parcellation of cortical ROIs used in the DMN analysis is provided in the Supplemental Material.

#### Statistical analysis

Independent sample two-tailed *t*-tests and $x^{2}$ tests were used to examine group differences in demographic factors, MRI quality metrics and neuropsychological measures. One ambidextrous SuperAging participant was excluded from the handedness $x^{2}\;$ analysis because no OACs were ambidextrous. The Pearson correlation was calculated between the time-series for each possible pair of ROI. For the 400-parcel atlas, this generated 79 800 unique coefficients which were averaged within-network to create seven average within-network coefficients for every participant. Average between-network connectivity was also computed for each participant (Supplemental Fig. 2). Average within-network FC was defined for each participant as the average of all coefficients between two ROIs of the same network (2618 coefficients per participant). For each participant, average between-network FC was defined as the average FC of all ROIs belonging to separate networks (77 182 coefficients per participant). Finally, system segregation was defined for each participant as the difference between within-network FC and between-network FC divided by within-network FC.

For subcomponents of the DMN, we generated 15 unique coefficients for every participant. Pearson’s coefficients were converted to Fisher’s *z*-transformed values for all analyses. After ensuring normality within groups,^82^ each within-network coefficient and DMN subcomponent *z*-value was compared across groups using two-sample independent *t*-tests with *α*’s adjusted for multiple comparisons using the Benjamini–Hochberg false discovery rate (FDR) at *q* = 0.05.^83^ All statistical analyses were performed within RStudio (version 2023.06.1 + 524).

## Results

### Data inclusion

At the time of our analysis, the SuperAging Research database included 92 participants with T1w scans and rs-fMRI scans from a Siemens 3T MAGNETOM TIM Trio scanner. Of those, 28 were excluded from our analysis due to unstable neuropsychological profiles across two research visits (*n* = 21) or unclear neuropsychological group profile (*n* = 7). An additional 24 were excluded due to artefacts in T1w data that made FreeSurfer segmentation fail (*n* = 6), contained rs-fMRI motion that surpassed FD thresholds (*n* = 17), or had insufficient useable imaging data (*n* = 1). Twenty-four SuperAgers and 16 OACs (*n* = 40) had MRI data and longitudinal neuropsychological profiles that met inclusion criteria for analysis in the present study (Fig. 1).

### Demographic and neuropsychological profiles

There were no significant between-group differences in demographic characteristics (all *P*-values > 0.05) including age, sex, handedness, education or time between research visits. Performance on neuropsychological measures was significantly different between SuperAgers and OACs for episodic memory as measured by the RAVLT delay (*t* = −11.85; *P* < 0.01), generative fluencies as measured by category fluency test (*t* = −2.49; *P* = 0.02) and confrontation naming as measured by the BNT-30 (*t* = −2.36; *P* = 0.03). The between-group difference in RAVLT delay performance is expected due to predefined group classification criteria. There were no significant between-group differences in the overall premorbid functional abilities as measured by the WTAR estimated Full-Scale Intelligence Quotient [FSIQ (*t* = −1.15; *P* = 0.26)] or executive functioning as measured by TMT-B (*t* = 1.38; *P* = 0.18). Statistical tests for group differences in demographic and neuropsychological profiles are summarized in Table 1.

### Functional connectivity analysis

After motion scrubbing, there were no significant between-group differences in mean FD or scan length. Group summary values are included in Table 1.

**Figure 4:**
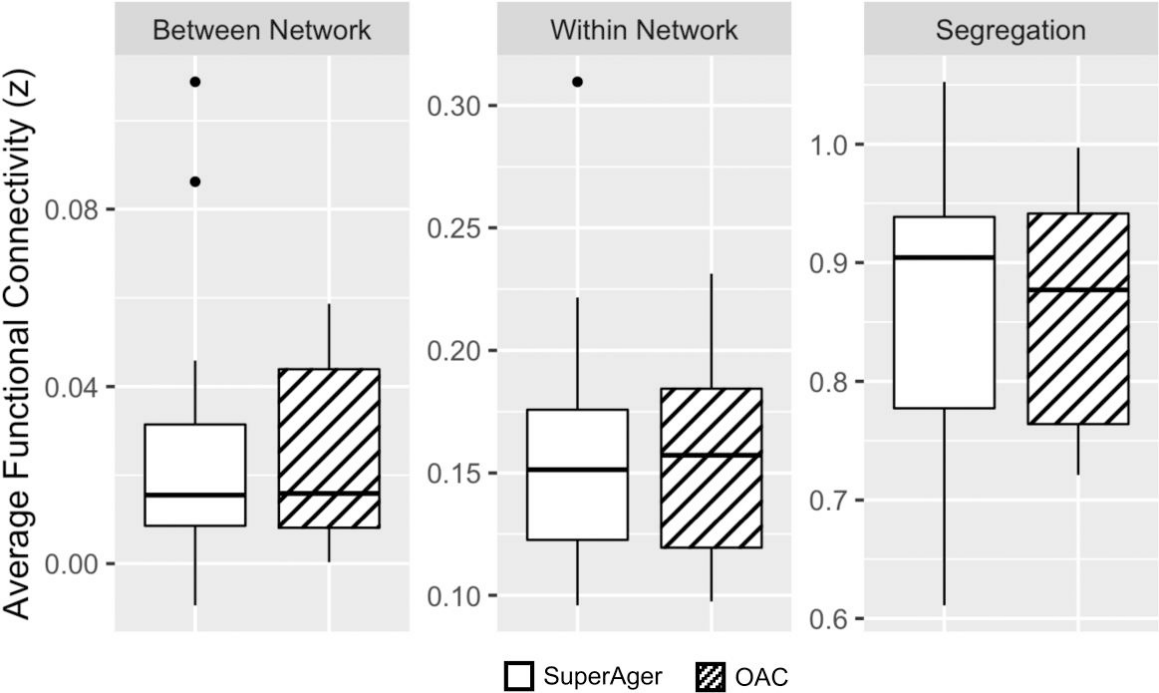
**Whole-brain measures of functional connectivity do not differ between SuperAgers and controls.** Network-wide average within-network functional connectivity and between-network connectivity did not differ between groups. A measure of network segregation (the difference between within-network FC and between-network FC divided by within-network FC) also did not differ between groups; OAC = older-aged controls. Wilcoxon signed-rank test; *P* > 0.05 for all comparisons.

Average within-network FC of seven resting-state networks were compared between SuperAgers and OACs. There were no significant differences between SuperAgers and OACs in within-network FC of the resting-state networks, including the DMN, executive control, limbic, ventral attention, dorsal attention, somatomotor and visual networks (*P*-values > FDR adjusted α; Fig. 2B). Similarly, there were no significant group differences in between-network FC (Supplemental Fig. 3). Additionally, broader measures of FC compared between SuperAgers and OACs, including average within-network FC from all networks, average between-network FC from all networks, and system segregation, did not differ significantly between groups (*P*-values > 0.05; Fig. 4).

FC between subregions of the DMN, including the IPL, PCC, MPC, PHC, MTG and hippocampus, were compared between SuperAgers and OACs. There were no significant between-group differences in FC of the four DMN ROIs after adjusting for FDR (*P*-values > FDR adjusted α; Fig. 3B). Notably, even before adjusting for FDR, we were unable to find significant between-group differences. Replication of all analyses without global signal regression similarly did not reveal significant group differences. Figures contain results from analyses with global signal regression.

## Discussion

The present study compared FC within seven canonical resting-state networks and between major regions of the DMN in SuperAgers and OACs. Results showed no significant group differences in FC between groups for any networks or regions of the DMN. The relationship between the large-scale resting-state networks and the spectrum of cognitive aging is complex and group-based average measurements of FC do not appear to explain the exceptional memory performance observed in SuperAgers. Potential contributors to these results, including the discrepancy between our findings and those that have found relatively strong within-network FC of successful cognitive agers,^34^ are likely multifactorial.

One possible contributor to the difference between our results and those of previous studies is our longitudinal inclusion criteria to reduce the risk of undiagnosed neurodegenerative processes among our participants. Of the 92 participants from the SuperAging Research Program with MRI considered for our study, 21 exhibited unstable neurocognitive profiles and were excluded. Participants with unstable neurocognitive profiles are not commonly accounted for in alternate successful cognitive aging rs-fMRI studies^59^ in part because the studies only have access to cognitive data from a single time-point. Participants with declining neurocognitive profiles could inadvertently drive differences in within- or between-network FC due to underlying ADRD. As such, group differences observed in prior studies may have been driven by ADRD. This highlights the importance of careful consideration of participant profiles in future studies aiming to elucidate the role of FC in cognitive aging.

It is also plausible that differences in rs-fMRI between SuperAgers and OACs are subtle and require highly precise measurements to detect. Subtle differences may be confounded by the relatively superior health of our OACs. Nonetheless, high-precision instruments are capable of identifying even the most nuanced group differences.^84,85^ For example, recent machine learning studies have successfully differentiated SuperAgers from OACs, albeit with limited generalizability due to sample overlap. Leveraging similar machine learning techniques in expanded datasets with more robust methods could potentially unveil significant and reliable findings. Furthermore, additional high-precision methods, such as adopting person-specific approaches for rs-fMRI metrics, may help capture subtle nuances that may differentiate SuperAgers from OACs.^35,86,87^

The present study of SuperAgers applies advanced neuroimaging methods, is the first to ensure participants maintain stable cognitive profiles and includes a cohort matched in size to similar studies^34,61,88^; however, it is limited in participant size. Nonetheless, at least one study^61^ was recently unable to detect previously reported group differences in FC of successful cognitive agers and controls observed with equivocal group size.^34^ Given the publication bias,^89^ there may also be unpublished studies with similar null results. Looking forward, the recent expansion of SuperAging Research Initiative (U19AG073153) into a multisite initiative will provide increased enrollment and greater power for future analyses. Future research may also make use of MRI scanners with higher magnetic field strength (e.g., 7 Tesla) or employ high-precision machine learning methods, both of which have been demonstrated to improve sensitivity^90,91^ and detect subtle group differences and have shown promise in recent studies.^62,63^

In conclusion, this study serves as a foundational step in exploring the complexity of large-scale neurocognitive networks and their relationship to cognitive aging. At the group-level, within-network FC of large-scale networks and between subcomponents of the DMN were not a primary differentiator between cognitively average aging and SuperAging phenotypes. Recognizing the complexity of this field, future research may benefit from considering the role of undiagnosed neurodegenerative processes and employing high-precision rs-fMRI measurements including those that allow for consideration of individual rather than group-based statistics. These efforts will undoubtedly enhance our understanding of the contributions of resting-state network integrity to cognitive aging trajectories and the factors that underlie the exceptional cognitive abilities of SuperAgers.

## Supplementary Material

fcae205_Supplementary_Data

## Data Availability

All codes used to conduct analyses are made available in the Supplemental Material. Atlases used for analyses are open source and publicly available: Schaefer cortical atlases (https://github.com/ThomasYeoLab/CBIG/) and HCP subcortical atlas (https://github.com/Washington-University/HCPpipelines/blob/master/global/templates/91282_Greyordinates/Atlas_ROIs.2.nii.gz). Data are available through a collaborative request to the SuperAging Research Initiative (superagingdata@uchicago.edu or emily.rogalski@bsd.uchicago.edu).
