# Supplementary material for: SuperAging functional connectomics from resting-state functional MRI
Source: Brain Commun. 2024 Jun 11;6(4):fcae205. doi: 10.1093/braincomms/fcae205 (PMC11228547; doi:10.1093/braincomms/fcae205)
Supplement: fcae205_Supplementary_Data [file fcae205_supplementary_data.zip › Supplementary_material.docx]

| ROI Name | Left Hemisphere Index | Right Hemisphere Index |
| --- | --- | --- |
| Inferior Parietal Lobe | 229 | 541 |
| Posterior Cingulate Cortex | 238 | 546 |
| Medial Prefrontal Cortex | 250 | 557 |
| Parahippocampal Cortex | 288 | 585 |
| Middle Temporal Gyrus | 258 | 563 |

**Supplemental Table 1 ROI Lookup Table Indices.** Lookup table indices for ROIs from Schaefer et al. (2018) used in rs-fMRI analysis^1^. The atlas is hosted on GitHub ( https://github.com/ThomasYeoLab/CBIG) and free for download; ROI=region of interest; rs-fMRI=resting state functional magnetic resonance imaging.


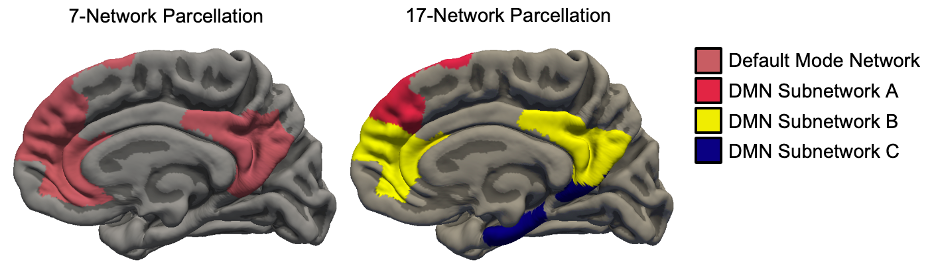


**Supplemental Figure 1 Absence of PHC ROI in the right hemisphere of the Schaefer/Yeo 7-network parcellation.** The default mode network (DMN) within the 7-network parcellation does not include the right PHC, while the more granular 17-network parcellation is comprised of multiple DMN subnetworks and DMN Subnetwork C includes the right PHC. The 17-network parcellation was used for the DMN subcomponent analysis.

**
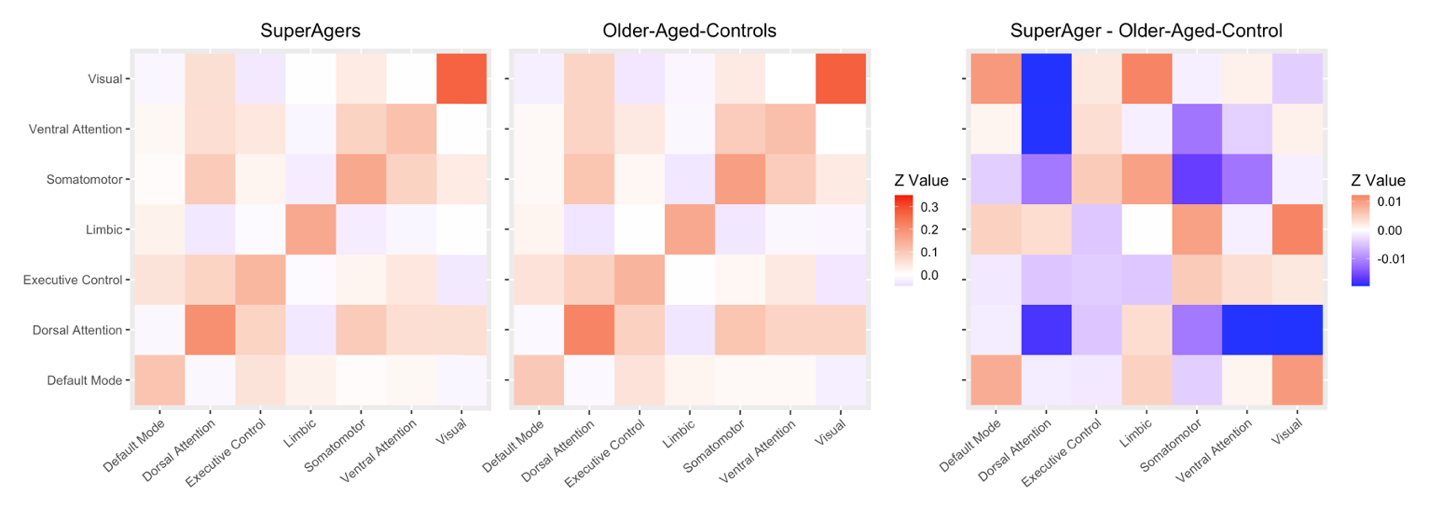
Supplemental Figure 2 Summary matrices of functional connectivity within and between networks show subtle differences between SuperAgers and Controls.** Adjacency matrices summarize within- (diagonal) and between- (off-diagonal) network functional connectity. The average adjacency matrix of SuperAgers (left) appears similar to that of the older-aged-controls (middle). Though no differences reached statistical significance, closer comparison (right) revealed subtle discrepancies between groups.

**
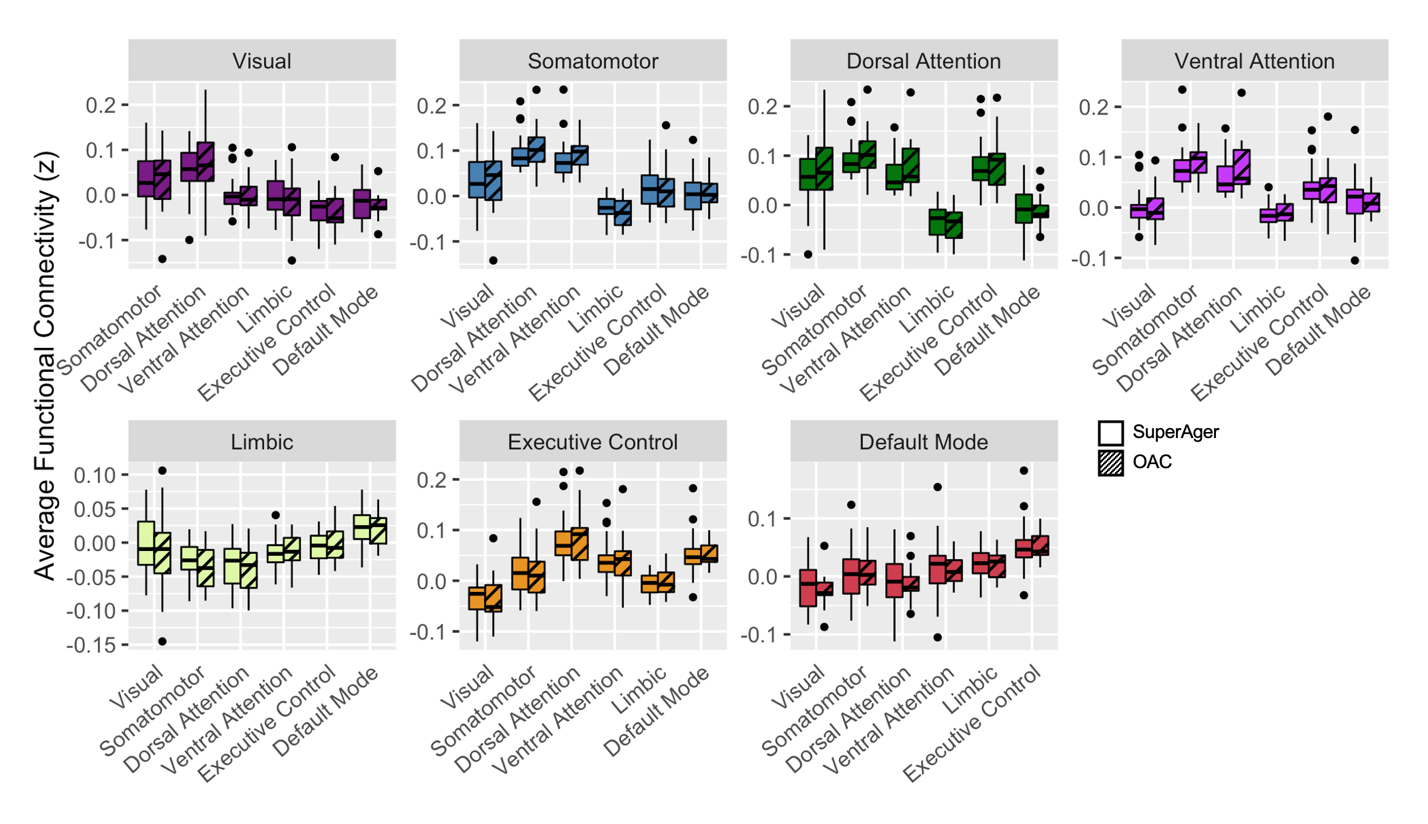
Supplemental Figure 3 Functional connectivity between networks did not significantly differ between SuperAgers and Controls.** Functional connectivity between networks did not differ between SuperAgers and Controls. Wilcoxon signed-rank test; P>0.05 for all comparisons.

# References

1. Schaefer A, Kong R, Gordon EM, Laumann TO, Zuo XN, Holmes AJ, et al. Local-Global Parcellation of the Human Cerebral Cortex from Intrinsic Functional Connectivity MRI. *Cereb Cortex.* 2018;**28**(9):3095-3114.
