# Supplementary material for: SuperAging functional connectomics from resting-state functional MRI
Source: Brain Commun. 2024 Jun 11;6(4):fcae205. doi: 10.1093/braincomms/fcae205 (PMC11228547; doi:10.1093/braincomms/fcae205)
Supplement: fcae205_Supplementary_Data [file fcae205_supplementary_data.zip › Supplementary_material_rsfMRI_Analysis.doc]

```
# Load in libraries
library(tidyverse)

# Set directories, read in subject list and group reference

data_release_dir <- "/Users/bramdiamond/Library/CloudStorage/Box-Box/Bram\ Shared\ Folder/Manuscripts/SA\ Resting\ State/Resubmission/Updated_Analysis"

subject_list <- read.csv(file.path(data_release_dir, "subjects_list.txt"), header = F, sep = ",", dec = ".")

group_ref <- read.table(file.path(data_release_dir, "group_ref.txt"), sep = "\t", header = F) %>%
  mutate(subject_id = V1,
         group = V2) %>%
  select(-V1, -V2)

# Install ggseg
# install.packages('ggseg')
library(ggseg)

# Install ggsegSchaefer
# Enable this universe
options(repos = c(
  ggseg = 'https://ggseg.r-universe.dev',
  CRAN = 'https://cloud.r-project.org'))

# install.packages('ggsegSchaefer')
library(ggsegSchaefer)

# DMN Analysis Networks -----------

# Install ggseg
# install.packages('ggseg')
library(ggseg)

# Install ggsegSchaefer
# Enable this universe
options(repos = c(
  ggseg = 'https://ggseg.r-universe.dev',
  CRAN = 'https://cloud.r-project.org'))

# DMN Visualizations -----------

# install.packages('ggsegSchaefer')

library(ggsegSchaefer)

rois <- schaefer17_600$data$label %>%
  as.data.frame() %>%
  filter(str_detect(., "Default")) %>%
  setNames(., "label") %>%
  mutate(color = case_when(label == "lh_17Networks_LH_DefaultA_IPL_2" ~ "DMN-IPL",
                           label == "lh_17Networks_LH_DefaultA_pCunPCC_4" ~ "DMN-PCC",
                           label == "lh_17Networks_LH_DefaultA_PFCm_4" ~ "DMN-PFC",
                           label == "lh_17Networks_LH_DefaultB_Temp_4" ~ "DMN-MTG",
                           label == "lh_17Networks_LH_DefaultC_PHC_1" ~ "DMN-PHC",
                           label == "rh_17Networks_RH_DefaultA_IPL_4" ~ "DMN-IPL",
                           label == "rh_17Networks_RH_DefaultA_pCunPCC_2" ~ "DMN-PCC",
                           label == "rh_17Networks_RH_DefaultA_PFCm_3" ~ "DMN-PFC",
                           label == "rh_17Networks_RH_DefaultB_Temp_1" ~ "DMN-MTG",
                           label == "rh_17Networks_RH_DefaultC_PHC_1" ~ "DMN-PHC",
                           TRUE ~ "DMN"))

my.cols <- RColorBrewer::brewer.pal(8, "Set1")
my.cols[1] <- "#CE3E4F"

rois %>%
  ggplot() +
  geom_brain(atlas = schaefer17_600,
             aes(fill = color),
             position = position_brain(hemi ~ side),
             colour = "black",
             size = 0.5,
             show.legend = T) +
  scale_fill_manual(values = my.cols, na.value = "white",
                    breaks = c("DMN", "DMN-IPL","DMN-PCC","DMN-PFC","DMN-MTG","DMN-PHC")) +
  theme_void() +
  theme(legend.position="right") +
  guides(fill = guide_legend(title = element_blank()))

aseg2 <- aseg

aseg2$data <- aseg$data %>%
  mutate(hippo = ifelse(region == "hippocampus", T, NA))

ggplot() +
  geom_brain(atlas = aseg2, side = "coronal", hemi = "right", aes(fill = hippo),
             colour = "black",
             size = 0.5,
             show.legend = T) +
  scale_fill_manual(values = my.cols[8], na.value="white") +
  theme_void() +
  theme(legend.position="right") +
  guides(fill = guide_legend(title = element_blank()))


# DMN Analysis without GSR -----------

all_dmn_cor_df_list = list()

for (i in 1:nrow(subject_list)) {

  # Assign sub ID
  sub <- subject_list[i,]

  # Location of avgwf
  surface_roi_avgwf_file <- file.path(data_release_dir, "Raw_Data", "noGSR", "dmn", "surface", list.files(path = file.path(data_release_dir, "Raw_Data", "noGSR", "dmn", "surface"),  pattern = sub))

  # Read in avgwf
  surface_roi_avgwf_table <- read.table(surface_roi_avgwf_file, sep = "\t", header = F) %>%
    t()

  colnames(surface_roi_avgwf_table) <- c("IPL", "PCC", "MPC", "MTG", "PHC")

  hippo_roi_avgwf_file <- file.path(data_release_dir, "Raw_Data", "noGSR", "dmn", "hippo", list.files(path = file.path(data_release_dir, "Raw_Data", "noGSR", "dmn", "hippo"),  pattern = sub))

  # Read in pconn
  hippo_roi_avgwf_table <- read.table(hippo_roi_avgwf_file, sep = "\t", header = F)

  colnames(hippo_roi_avgwf_table) <- c("Hippo")

  dmn_avgwf_df <- surface_roi_avgwf_table %>%
    cbind(hippo_roi_avgwf_table)

  dmn_cor_matrix <- cor(dmn_avgwf_df)

  # dmn_cor_matrix[upper.tri(dmn_cor_matrix, diag = TRUE)] <- NA

  # Install reshape2 for melt() function
  # install.packages('reshape2')
  library(reshape2)

  # Turn correlation matrix into long dataframe
  dmn_cor_df <- melt(as.matrix(dmn_cor_matrix), value.name = "r_value") %>%
    # Remove NA values (i.e. the top triangle and diagonal)
    drop_na()

  # Convert r values to Fisher's z-scores
  # Install correlation package
  # install.packages('correlation')
  library(correlation)

  dmn_cor_df <- dmn_cor_df %>%
    mutate(z_value = z_fisher(r = r_value)) %>%
    select(-r_value)

  # Finally, add subject id
  dmn_cor_df <- dmn_cor_df %>%
    mutate(subject_id = sub)

  # Add cor dataframe to growing list of all subject pconn dataframes
  all_dmn_cor_df_list[i] <- list(dmn_cor_df)

}

# Bind all_pconn_dataframes; turn it from a list to a dataframe
all_dmn_cor_df <- bind_rows(all_dmn_cor_df_list) %>%
  # Remove ROIs pairs that are the same
  subset(., Var1 != Var2) %>%
  # Add groups
  left_join(., group_ref, by = join_by(subject_id)) %>%
  # Change names to SuperAger and OAC (older-aged control)
  mutate(group = case_when(group == "Normal" ~ "OAC",
                           group == "Super" ~ "SuperAger"))

# Test for group differences and visualize results
# Install ggpubr
# install.packages('ggpubr')
library(ggpubr)

# install.packages("remotes")
# remotes::install_github("coolbutuseless/ggpattern")
library(ggpattern)

all_dmn_cor_df %>%
  group_by(group, Var1, Var2) %>%
  # Factor and reorder group and networks (this makes the graphs look nicer)
  mutate(group = factor(group, levels = c('SuperAger', 'OAC')),
         Var1 = factor(Var1, levels = c("IPL","PCC","MPC","MTG","PHC", "Hippo")),
         Var2 = factor(Var2, levels = c("IPL","PCC","MPC","MTG","PHC", "Hippo"))) %>%
  # Test and plot
  ggplot(aes(y = z_value, x = Var2, pattern = group)) +
  facet_wrap(~Var1, scales = "free_x", nrow = 2) +
  geom_boxplot_pattern(position = "dodge", aes(fill = Var1), color = "black", pattern_color = "black", pattern_fill = "black", pattern_angle = 45, pattern_density = 0.1, pattern_spacing = 0.03, pattern_key_scale_factor = 0.3) +
  scale_pattern_manual(values = c(SuperAger = "none", OAC = "stripe")) +
  scale_fill_manual(values = c(my.cols[2:6], my.cols[8])) +
  # # Run stats
  stat_compare_means(aes(group = group),
                     paired = F,
                     method = "wilcox.test",
                     label = "..p.format..") +
  labs(
    pattern = "Group",
    y = "Average Functional Connectivity (z)"
  ) +
  guides(
    fill = "none",
  ) +
  theme(
    axis.ticks.x = element_blank(),
    axis.title.x = element_blank()
  )  +
 scale_y_continuous(expand = expansion(add = 0.2))

# Without p-values

all_dmn_cor_df %>%
  group_by(group, Var1, Var2) %>%
  # Factor and reorder group and networks (this makes the graphs look nicer)
  mutate(group = factor(group, levels = c('SuperAger', 'OAC')),
         Var1 = factor(Var1, levels = c("IPL","PCC","MPC","MTG","PHC", "Hippo")),
         Var2 = factor(Var2, levels = c("IPL","PCC","MPC","MTG","PHC", "Hippo"))) %>%
  # Test and plot
  ggplot(aes(y = z_value, x = Var2, pattern = group)) +
  facet_wrap(~Var1, scales = "free_x", nrow = 2) +
  geom_boxplot_pattern(position = "dodge", aes(fill = Var1), color = "black", pattern_color = "black", pattern_fill = "black", pattern_angle = 45, pattern_density = 0.1, pattern_spacing = 0.03, pattern_key_scale_factor = 0.3) +
  scale_pattern_manual(values = c(SuperAger = "none", OAC = "stripe")) +
  scale_fill_manual(values = c(my.cols[2:6], my.cols[8])) +
  labs(
    pattern = "Group",
    y = "Average Functional Connectivity (z)"
  ) +
  guides(
    fill = "none",
  ) +
  theme(
    axis.ticks.x = element_blank(),
    axis.title.x = element_blank()
  )


# DMN Analysis with GSR -----------

all_dmn_cor_df_list = list()

for (i in 1:nrow(subject_list)) {

  # Assign sub ID
  sub <- subject_list[i,]

  # Location of pconn
  surface_roi_avgwf_file <- file.path(data_release_dir, "Raw_Data", "GSR", "dmn", "surface", list.files(path = file.path(data_release_dir, "Raw_Data", "GSR", "dmn", "surface"),  pattern = sub))

  # Read in pconn
  surface_roi_avgwf_table <- read.table(surface_roi_avgwf_file, sep = "\t", header = F) %>%
    t()

  colnames(surface_roi_avgwf_table) <- c("IPL", "PCC", "MPC", "MTG", "PHC")

  hippo_roi_avgwf_file <- file.path(data_release_dir, "Raw_Data", "noGSR", "dmn", "hippo", list.files(path = file.path(data_release_dir, "Raw_Data", "noGSR", "dmn", "hippo"),  pattern = sub))

  # Read in pconn
  hippo_roi_avgwf_table <- read.table(hippo_roi_avgwf_file, sep = "\t", header = F)

  colnames(hippo_roi_avgwf_table) <- c("Hippo")

  dmn_avgwf_df <- surface_roi_avgwf_table %>%
    cbind(hippo_roi_avgwf_table)

  dmn_cor_matrix <- cor(dmn_avgwf_df)

  # dmn_cor_matrix[upper.tri(dmn_cor_matrix, diag = TRUE)] <- NA

  # Install reshape2 for melt() function
  # install.packages('reshape2')
  library(reshape2)

  # Turn correlation matrix into long dataframe
  dmn_cor_df <- melt(as.matrix(dmn_cor_matrix), value.name = "r_value") %>%
    # Remove NA values (i.e. the top triangle and diagonal)
    drop_na()

  # Convert r values to Fisher's z-scores
  # Install correlation package
  # install.packages('correlation')
  library(correlation)

  dmn_cor_df <- dmn_cor_df %>%
    mutate(z_value = z_fisher(r = r_value)) %>%
    select(-r_value)

  # Finally, add subject id
  dmn_cor_df <- dmn_cor_df %>%
    mutate(subject_id = sub)

  # Add pconn dataframe to growing list of all subject pconn dataframes
  all_dmn_cor_df_list[i] <- list(dmn_cor_df)

}

# Bind all_pconn_dataframes; turn it from a list to a dataframe
all_dmn_cor_df <- bind_rows(all_dmn_cor_df_list) %>%
  # Remove ROIs pairs that are the same
  subset(., Var1 != Var2) %>%
  # Add groups
  left_join(., group_ref, by = join_by(subject_id)) %>%
  # Change names to SuperAger and OAC (older-aged control)
  mutate(group = case_when(group == "Normal" ~ "OAC",
                           group == "Super" ~ "SuperAger"))

# Test for group differences and visualize results
# Install ggpubr
# install.packages('ggpubr')
library(ggpubr)

# install.packages("remotes")
# remotes::install_github("coolbutuseless/ggpattern")
library(ggpattern)

all_dmn_cor_df %>%
  group_by(group, Var1, Var2) %>%
  # Factor and reorder group and networks (this makes the graphs look nicer)
  mutate(group = factor(group, levels = c('SuperAger', 'OAC')),
         Var1 = factor(Var1, levels = c("IPL","PCC","MPC","MTG","PHC", "Hippo")),
         Var2 = factor(Var2, levels = c("IPL","PCC","MPC","MTG","PHC", "Hippo"))) %>%
  # Test and plot
  ggplot(aes(y = z_value, x = Var2, pattern = group)) +
  facet_wrap(~Var1, scales = "free_x", nrow = 2) +
  geom_boxplot_pattern(position = "dodge", aes(fill = Var1), color = "black", pattern_color = "black", pattern_fill = "black", pattern_angle = 45, pattern_density = 0.1, pattern_spacing = 0.03, pattern_key_scale_factor = 0.1) +
  scale_pattern_manual(values = c(SuperAger = "none", OAC = "stripe")) +
  scale_fill_manual(values = c(my.cols[2:6], my.cols[8])) +
  # # Run stats
  stat_compare_means(aes(group = group),
                     paired = F,
                     method = "wilcox.test",
                     label = "..p.format..") +
  labs(
    pattern = "Group",
    y = "Average Functional Connectivity (z)"
  ) +
  guides(
    fill = "none",
  ) +
  theme(
    axis.ticks.x = element_blank(),
    axis.title.x = element_blank()
  )  +
  scale_y_continuous(expand = expansion(add = 0.2))

# Without P-Values
all_dmn_cor_df %>%
  group_by(group, Var1, Var2) %>%
  # Factor and reorder group and networks (this makes the graphs look nicer)
  mutate(group = factor(group, levels = c('SuperAger', 'OAC')),
         Var1 = factor(Var1, levels = c("IPL","PCC","MPC","MTG","PHC", "Hippo")),
         Var2 = factor(Var2, levels = c("IPL","PCC","MPC","MTG","PHC", "Hippo"))) %>%
  # Test and plot
  ggplot(aes(y = z_value, x = Var2, pattern = group)) +
  facet_wrap(~Var1, scales = "free", nrow = 2) +
  geom_boxplot_pattern(position = "dodge", aes(fill = Var1), color = "black", pattern_color = "black", pattern_fill = "black", pattern_angle = 45, pattern_density = 0.1, pattern_spacing = 0.03, pattern_key_scale_factor = 0.1) +
  scale_pattern_manual(values = c(SuperAger = "none", OAC = "stripe")) +
  scale_fill_manual(values = c(my.cols[2:6], my.cols[8])) +
  theme_grey(base_size = 15) +
  labs(
    pattern = "Group",
    y = "Average Functional Connectivity (z)"
  ) +
  guides(
    fill = "none",
  ) +
  theme(
    axis.ticks.x = element_blank(),
    axis.title.x = element_blank()
  )  +
  scale_y_continuous(expand = expansion(add = 0.2))

# For graphical abstract

# Without P-Values
all_dmn_cor_df %>%
  group_by(group, Var1, Var2) %>%
  # Factor and reorder group and networks (this makes the graphs look nicer)
  mutate(group = factor(group, levels = c('SuperAger', 'OAC')),
         Var1 = factor(Var1, levels = c("IPL","PCC","MPC","MTG","PHC", "Hippo")),
         Var2 = factor(Var2, levels = c("IPL","PCC","MPC","MTG","PHC", "Hippo"))) %>%
  # Test and plot
  ggplot(aes(y = z_value, x = Var2, pattern = group)) +
  facet_wrap(~Var1, scales = "free", nrow = 2) +
  geom_boxplot_pattern(position = "dodge", aes(fill = Var1), color = "black", pattern_color = "black", pattern_fill = "black", pattern_angle = 45, pattern_density = 0.1, pattern_spacing = 0.03, pattern_key_scale_factor = 0.1) +
  scale_pattern_manual(values = c(SuperAger = "none", OAC = "stripe")) +
  scale_fill_manual(values = c(my.cols[2:6], my.cols[8])) +
  theme_grey(base_size = 15) +
  theme_classic() +
  labs(
    pattern = "Group",
    y = "Average Functional Connectivity (z)"
  ) +
  guides(
    fill = "none",
  ) +
  theme(
    axis.ticks.x = element_blank(),
    axis.title.x = element_blank()
  )  +
  scale_y_continuous(expand = expansion(add = 0.2))


# 7 Network Analysis -----------
# 7 Network Visualizations -----------
rois <- schaefer7_400$data$label %>%
  as.data.frame() %>%
  setNames(., "label")

rois <- data.frame(rois) %>%
  mutate(color = case_when(grepl("Vis", label) ~ "Visual Network",
                           grepl("SomMot", label) ~ "Somatomotor Network",
                           grepl("DorsAttn", label) ~ "Dorsal Attention Network",
                           grepl("SalVentAttn", label) ~ "Ventral Attention Network",
                           grepl("Limbic", label) ~ "Limbic Network",
                           grepl("Cont", label) ~ "Executive Control Network",
                           grepl("Default", label) ~ "Default Mode Network")) %>%
  mutate(color = factor(color, levels = c("Visual Network",
                                          "Somatomotor Network",
                                          "Dorsal Attention Network",
                                          "Ventral Attention Network",
                                          "Limbic Network",
                                          "Executive Control Network",
                                          "Default Mode Network")))

my.cols <- c("#791286",
             "#4882B4",
             "#02760F",
             "#C639FB",
             "#DEF8A7",
             "#E89423",
             "#CE3E4F")

cortical_pos <- c("left lateral", "left medial", "right medial", "right lateral")

rois %>%
  ggplot() +
  geom_brain(atlas = schaefer7_400,
             aes(fill = color),
             position = position_brain(hemi ~ side),
             colour = "black",
             size = 0.5,
             show.legend = T) +
  scale_fill_manual(values = my.cols, na.value="white",
                    breaks = c("Visual Network",
                               "Somatomotor Network",
                               "Dorsal Attention Network",
                               "Ventral Attention Network",
                               "Limbic Network",
                               "Executive Control Network",
                               "Default Mode Network")) +
  labs(
    fill = element_blank()
  ) +
  theme_void() +
  theme(legend.position = "right")

# 7 Network Analysis without GSR -----------

schaefer_407_info <- read.table(file.path(data_release_dir, "Schaefer2018_400Parcels_7Networks_order_info.txt"), sep = "\t", header = F)


# Clean up dataframe
schaefer_407_info <- schaefer_407_info %>%
  # Separate parcel info and color
  mutate(column_name = rep(c("parcel_id_info", "parcel_color"), nrow(schaefer_407_info) / 2),
         key = rep(1:(nrow(schaefer_407_info) / 2), each = 2)) %>%
  pivot_wider(id_cols = key, names_from = column_name, values_from = V1) %>%
  select(-key) %>%
  # Clean up "parcel_id_info" column
  separate_wider_delim(cols = parcel_id_info, delim = "_", names = c("atlas", "hemi", "parc_name"), too_many = "merge") %>%
  # Clean up "parcel_color" column
  separate_wider_delim(cols = parcel_color, delim = " ", names = c("parcel_id", "r", "g", "b", "alpha")) %>%
  # Make network name column
  mutate(net_name = parc_name) %>%
  separate_wider_delim(cols = net_name, delim = "_", names = c("net_name"), too_many = "drop") %>%
  # Make full network name column
  mutate(network_name = case_when(net_name == "Vis" ~ "Visual",
                                  net_name == "SomMot" ~ "Somatomotor",
                                  net_name == "DorsAttn" ~ "Dorsal Attention",
                                  net_name == "SalVentAttn" ~ "Ventral Attention",
                                  net_name == "Limbic" ~ "Limbic",
                                  net_name == "Cont" ~ "Executive Control",
                                  net_name == "Default" ~ "Default Mode")) %>%
  # Replace r b g with hex (and remove alpha); first average r, g, b within network because they are slightly different
  group_by(network_name) %>%
  reframe(m_r = mean(as.numeric(r)),
            m_g = mean(as.numeric(g)),
            m_b = mean(as.numeric(b)),
            across()) %>%
  mutate(rgb = rgb(m_r, m_g, m_b, maxColorValue = 255)) %>%
  select(-r, -g, -b, -m_r, -m_g, -m_b, -alpha) %>%
  # Finally, factor these color; this is helpful for visualization
  mutate(rgb = factor(rgb, levels = c("#791286",
                                      "#4882B4",
                                      "#02760F",
                                      "#C639FB",
                                      "#DEF8A7",
                                      "#E89423",
                                      "#CE3E4F")))

# Create summary dataframe of correlation matrices (pconn)

all_pconn_dataframes_list = list()

for (i in 1:nrow(subject_list)) {

  # Assign sub ID
  sub <- subject_list[i,]

  # Location of pconn
  pconn_file <- file.path(data_release_dir, "Raw_Data/noGSR/7network", list.files(path = file.path(data_release_dir, "Raw_Data/noGSR/7network"),  pattern = sub))

  # Read in pconn
  pconn <- read.table(pconn_file, sep = "\t", header = F)

  # # Remove the top triangle (and diagonal) of the pconn
  # pconn[upper.tri(as.matrix(pconn), diag = T)] = NA

  # Make column name column number (i.e., parcel_id)
  colnames(pconn) <- c(1:400)

  # Install reshape2 for melt() function
  # install.packages('reshape2')
  library(reshape2)

  # Turn correlation matrix into long dataframe
  pconn_dataframe <- melt(as.matrix(pconn), value.name = "r_value") %>%
    # Remove NA values (i.e. the top triangle and diagonal)
    drop_na()

  # Convert r values to Fisher's z-scores
  # Install correlation package
  # install.packages('correlation')
  library(correlation)

  pconn_dataframe <- pconn_dataframe %>%
    mutate(z_value = z_fisher(r = r_value)) %>%
    select(-r_value)

  # Finally, add subject id
  pconn_dataframe <- pconn_dataframe %>%
    mutate(subject_id = sub)

  # Add pconn dataframe to growing list of all subject pconn dataframes
  all_pconn_dataframes_list[i] <- list(pconn_dataframe)

}

# Bind all_pconn_dataframes; turn it from a list to a dataframe
all_pconn_dataframes <- bind_rows(all_pconn_dataframes_list)

# Within-network analysis


# Summarize dataframe into within-network functional connectivity (i.e., z_value); summarize for each network and for each subject

# First, add network names for each parcel involved

all_pconn_dataframes <- all_pconn_dataframes %>%
  # Make sure Var1 and Var2 are characters (they are technically parcel_id)
  mutate(Var1 = as.character(Var1),
         Var2 = as.character(Var2)) %>%
  # Add columns for networks associated with Var1 and Var2; this is taken from schaefer_407_info defined above
  left_join(., schaefer_407_info %>% select(parcel_id, network_name), by = c("Var1" = "parcel_id")) %>%
  # Change name to reflext network for Var1
  mutate(Var1_network_name = network_name) %>%
  select(-network_name) %>%
  # Do the same for Var2
  left_join(., schaefer_407_info %>% select(parcel_id, network_name), by = c("Var2" = "parcel_id")) %>%
  # Change name to reflext network for Var1
  mutate(Var2_network_name = network_name) %>%
  select(-network_name)

# 7 Network Summary Correlation Matrices -----------

# SuperAgers
all_pconn_dataframes %>%
  left_join(., group_ref) %>%
  # Change names to SuperAger and OAC (older-aged control)
  mutate(group = case_when(group == "Normal" ~ "OAC",
                           group == "Super" ~ "SuperAger")) %>%
  filter(group == "SuperAger") %>%
  group_by(Var1_network_name, Var2_network_name) %>%
  filter(!is.infinite(z_value)) %>%
  summarise(z_value = mean(z_value)) %>%
  ggplot(aes(x = Var1_network_name, y = Var2_network_name, fill = z_value)) +
  geom_raster() +
  theme_bw() +
  # Because we need the x and y axis to display every node,
  # not just the nodes that have connections to each other,
  # make sure that ggplot does not drop unused factor levels
  scale_x_discrete(drop = FALSE) +
  scale_y_discrete(drop = FALSE) +
  theme_grey(base_size = 15) +
  labs(
    title = "SuperAgers"
  ) +
  theme(
    # Rotate the x-axis lables so they are legible
    axis.text.x = element_text(angle=40, vjust=1, hjust=1),
    axis.title = element_blank(),
    plot.title = element_text(hjust = 0.5),
    # Force the plot into a square aspect ratio
    aspect.ratio = 1) +
    scale_fill_gradient2(limits = c(0, 0.65), midpoint = 0.15, low = "white", high = "red") +
  labs(
    fill = "Z Value"
  )

# OAC
all_pconn_dataframes %>%
  left_join(., group_ref) %>%
  # Change names to SuperAger and OAC (older-aged control)
  mutate(group = case_when(group == "Normal" ~ "Older-Aged-Control",
                           group == "Super" ~ "SuperAger")) %>%
  filter(group == "Older-Aged-Control") %>%
  group_by(Var1_network_name, Var2_network_name) %>%
  filter(!is.infinite(z_value)) %>%
  summarise(z_value = mean(z_value)) %>%
  ggplot(aes(x = Var1_network_name, y = Var2_network_name, fill = z_value)) +
  geom_raster() +
  theme_bw() +
  # Because we need the x and y axis to display every node,
  # not just the nodes that have connections to each other,
  # make sure that ggplot does not drop unused factor levels
  scale_x_discrete(drop = FALSE) +
  scale_y_discrete(drop = FALSE) +
  theme_grey(base_size = 15) +
  labs(
    title = "Older-Aged-Controls"
  ) +
  theme(
    # Rotate the x-axis lables so they are legible
    axis.text.x = element_text(angle=40, vjust=1, hjust=1),
    axis.title = element_blank(),
    plot.title = element_text(hjust = 0.5),
    # Force the plot into a square aspect ratio
    aspect.ratio = 1) +
  scale_fill_gradient2(limits = c(0, 0.65), midpoint = 0.15, low = "white", high = "red") +
  labs(
    fill = "Z Value"
  )

# 7 Network Group Difference Correlation Matrices -----------

all_pconn_dataframes %>%
  left_join(., group_ref) %>%
  # Change names to SuperAger and OAC (older-aged control)
  mutate(group = case_when(group == "Normal" ~ "OAC",
                           group == "Super" ~ "SuperAger")) %>%
  group_by(group, Var1_network_name, Var2_network_name) %>%
  filter(!is.infinite(z_value)) %>%
  summarise(z_value = mean(z_value)) %>%
  pivot_wider(names_from = group, values_from = z_value) %>%
  mutate(diff = SuperAger - OAC) %>%
  ggplot(aes(x = Var1_network_name, y = Var2_network_name, fill = diff)) +
  geom_raster() +
  theme_bw() +
  # Because we need the x and y axis to display every node,
  # not just the nodes that have connections to each other,
  # make sure that ggplot does not drop unused factor levels
  scale_x_discrete(drop = FALSE) +
  scale_y_discrete(drop = FALSE) +
  theme_grey(base_size = 15) +
  labs(
    title = "SuperAger - Older-Aged-Control"
  ) +
  theme(
    # Rotate the x-axis lables so they are legible
    axis.text.x = element_text(angle=40, vjust=1, hjust=1),
    axis.title = element_blank(),
    plot.title = element_text(hjust = 0.5),
    # Force the plot into a square aspect ratio
    aspect.ratio = 1) +
  scale_fill_gradient2(midpoint = 0.01, low = "white", high = "red") +
  labs(
    fill = "Z Value"
  )
# 7 Network Within Networks -----------

all_pconn_dataframes %>%
  filter(!is.infinite(z_value),
         Var1_network_name == Var2_network_name) %>%
  group_by(subject_id, Var1_network_name) %>%
  summarise(avg_z_value = mean(z_value)) %>%
  ungroup() %>%
  left_join(., group_ref) %>%
  # Change names to SuperAger and OAC (older-aged control)
  mutate(group = case_when(group == "Normal" ~ "OAC",
                           group == "Super" ~ "SuperAger")) %>%
  rename(network_name = Var1_network_name) %>%
  # Factor and reorder group and networks (this makes the graphs look nicer)
  mutate(group = factor(group, levels = c('SuperAger', 'OAC')),
         network_name = factor(network_name, levels = c("Visual",
                                                        "Somatomotor",
                                                        "Dorsal Attention",
                                                        "Ventral Attention",
                                                        "Limbic",
                                                        "Executive Control",
                                                        "Default Mode"))) %>%
  # Test and plot
  ggplot(aes(y = avg_z_value, x = group, pattern = group)) +
  facet_wrap(~network_name, scales = "free_y", nrow = 2) +
  # Run stats
  stat_compare_means(comparisons = list(c("SuperAger", "OAC")),
                     paired = F,
                     method = "wilcox.test",
                     label = "..p.format..") +
  geom_boxplot_pattern(position = "dodge", aes(fill = network_name), color = "black", pattern_color = "black", pattern_fill = "black", pattern_angle = 45, pattern_density = 0.1, pattern_spacing = 0.06, pattern_key_scale_factor = 0.1) +
  # Get colors from schaefer_407_info
  theme_grey(base_size = 15) +
  scale_fill_manual(values = c(as.character(sort(unique(schaefer_407_info$rgb))))) +
  scale_pattern_manual(values = c(SuperAger = "none", OAC = "stripe"),) +
  labs(
    fill = "Resting State Network",
    y = "Average Functional Connectivity (z)",
    pattern = "Group"
  ) +
  theme(
    axis.ticks.x = element_blank(),
    axis.title.x = element_blank(),
    axis.text.x = element_blank()
  ) +
  scale_y_continuous(expand = expansion(add = 0.05))

# Without P-Values

all_pconn_dataframes %>%
  filter(!is.infinite(z_value),
         Var1_network_name == Var2_network_name) %>%
  group_by(subject_id, Var1_network_name) %>%
  summarise(avg_z_value = mean(z_value)) %>%
  ungroup() %>%
  left_join(., group_ref) %>%
  # Change names to SuperAger and OAC (older-aged control)
  mutate(group = case_when(group == "Normal" ~ "OAC",
                           group == "Super" ~ "SuperAger")) %>%
  rename(network_name = Var1_network_name) %>%
  # Factor and reorder group and networks (this makes the graphs look nicer)
  mutate(group = factor(group, levels = c('SuperAger', 'OAC')),
         network_name = factor(network_name, levels = c("Visual",
                                                        "Somatomotor",
                                                        "Dorsal Attention",
                                                        "Ventral Attention",
                                                        "Limbic",
                                                        "Executive Control",
                                                        "Default Mode"))) %>%
  # Test and plot
  ggplot(aes(y = avg_z_value, x = group, pattern = group)) +
  facet_wrap(~network_name, scales = "free_y", nrow = 2) +
  geom_boxplot_pattern(position = "dodge", aes(fill = network_name), color = "black", pattern_color = "black", pattern_fill = "black", pattern_angle = 45, pattern_density = 0.1, pattern_spacing = 0.06, pattern_key_scale_factor = 0.1) +
  # Get colors from schaefer_407_info
  theme_grey(base_size = 15) +
  scale_fill_manual(values = c(as.character(sort(unique(schaefer_407_info$rgb))))) +
  scale_pattern_manual(values = c(SuperAger = "none", OAC = "stripe"),) +
  labs(
    fill = "Resting State Network",
    y = "Average Functional Connectivity (z)",
    pattern = "Group"
  ) +
  theme(
    axis.ticks.x = element_blank(),
    axis.title.x = element_blank(),
    axis.text.x = element_blank()
  )

# 7 Network Between Networks -----------

all_pconn_dataframes %>%
  filter(!is.infinite(z_value),
         Var1_network_name != Var2_network_name) %>%
  group_by(subject_id, Var1_network_name, Var2_network_name) %>%
  summarise(avg_z_value = mean(z_value)) %>%
  ungroup() %>%
  left_join(., group_ref) %>%
  # Change names to SuperAger and OAC (older-aged control)
  mutate(group = case_when(group == "Normal" ~ "OAC",
                           group == "Super" ~ "SuperAger")) %>%
  # Factor and reorder group and networks (this makes the graphs look nicer)
  mutate(group = factor(group, levels = c('SuperAger', 'OAC')),
         Var1_network_name = factor(Var1_network_name, levels = c("Visual",
                                                        "Somatomotor",
                                                        "Dorsal Attention",
                                                        "Ventral Attention",
                                                        "Limbic",
                                                        "Executive Control",
                                                        "Default Mode")),
         Var2_network_name = factor(Var2_network_name, levels = c("Visual",
                                                        "Somatomotor",
                                                        "Dorsal Attention",
                                                        "Ventral Attention",
                                                        "Limbic",
                                                        "Executive Control",
                                                        "Default Mode"))) %>%
  # Test and plot
  ggplot(aes(y = avg_z_value, x = Var2_network_name, pattern = group)) +
  facet_wrap(~Var1_network_name, scales = "free", nrow = 2) +
  # Run stats
  stat_compare_means(aes(group = group),
                     paired = F,
                     method = "wilcox.test",
                     label = "..p.format..") +
  geom_boxplot_pattern(position = "dodge", aes(fill = Var1_network_name), color = "black", pattern_color = "black", pattern_fill = "black", pattern_angle = 45, pattern_density = 0.1, pattern_spacing = 0.06, pattern_key_scale_factor = 0.1) +
  # Get colors from schaefer_407_info
  theme_grey(base_size = 15) +
  scale_fill_manual(values = c(as.character(sort(unique(schaefer_407_info$rgb))))) +
  scale_pattern_manual(values = c(SuperAger = "none", OAC = "stripe"),) +
  labs(
    fill = "Resting State Network",
    y = "Average Functional Connectivity (z)",
    pattern = "Group"
  ) +
  theme(
    axis.ticks.x = element_blank(),
    axis.title.x = element_blank(),
    axis.text.x = element_text(angle=40, vjust=1, hjust=1)
  ) +
  scale_y_continuous(expand = expansion(add = 0.1))

# No P-Values

all_pconn_dataframes %>%
  filter(!is.infinite(z_value),
         Var1_network_name != Var2_network_name) %>%
  group_by(subject_id, Var1_network_name, Var2_network_name) %>%
  summarise(avg_z_value = mean(z_value)) %>%
  ungroup() %>%
  left_join(., group_ref) %>%
  # Change names to SuperAger and OAC (older-aged control)
  mutate(group = case_when(group == "Normal" ~ "OAC",
                           group == "Super" ~ "SuperAger")) %>%
  # Factor and reorder group and networks (this makes the graphs look nicer)
  mutate(group = factor(group, levels = c('SuperAger', 'OAC')),
         Var1_network_name = factor(Var1_network_name, levels = c("Visual",
                                                                  "Somatomotor",
                                                                  "Dorsal Attention",
                                                                  "Ventral Attention",
                                                                  "Limbic",
                                                                  "Executive Control",
                                                                  "Default Mode")),
         Var2_network_name = factor(Var2_network_name, levels = c("Visual",
                                                                  "Somatomotor",
                                                                  "Dorsal Attention",
                                                                  "Ventral Attention",
                                                                  "Limbic",
                                                                  "Executive Control",
                                                                  "Default Mode"))) %>%
  # Test and plot
  ggplot(aes(y = avg_z_value, x = Var2_network_name, pattern = group)) +
  facet_wrap(~Var1_network_name, scales = "free", nrow = 2) +
  geom_boxplot_pattern(position = "dodge", aes(fill = Var1_network_name), color = "black", pattern_color = "black", pattern_fill = "black", pattern_angle = 45, pattern_density = 0.1, pattern_spacing = 0.06, pattern_key_scale_factor = 0.1) +
  # Get colors from schaefer_407_info
  theme_grey(base_size = 15) +
  scale_fill_manual(values = c(as.character(sort(unique(schaefer_407_info$rgb))))) +
  scale_pattern_manual(values = c(SuperAger = "none", OAC = "stripe"),) +
  labs(
    fill = "Resting State Network",
    y = "Average Functional Connectivity (z)",
    pattern = "Group"
  ) +
  theme(
    axis.ticks.x = element_blank(),
    axis.title.x = element_blank(),
    axis.text.x = element_text(angle=40, vjust=1, hjust=1)
  )

# 7 Network Average Within/Between/Segregation -----------

# Summarize dataframe into between-network functional connectivity (i.e., z_value); summarize for each subject

# Second, summarize across networks within-network and between-network averages for each subject


all_across_networks_summary <- all_pconn_dataframes %>%
  filter(!is.infinite(z_value)) %>%
  # Add column to check if networks are the same
  mutate(within_network = ifelse(Var1_network_name == Var2_network_name, "Within Network", "Between Network")) %>%
  # Average within subjects
  group_by(subject_id, within_network) %>%
  summarise(z_value = mean(z_value)) %>%
  ungroup() %>%
  # Calculate segregation value
  pivot_wider(names_from = within_network, values_from = z_value) %>%
  mutate(Segregation = (`Within Network` - `Between Network`) / `Within Network`) %>%
  pivot_longer(cols = c(`Within Network`, `Between Network`, Segregation),
               names_to = "measure",
               values_to = "value") %>%
  left_join(., group_ref, by = join_by(subject_id)) %>%
  # Change names to SuperAger and OAC (older-aged control)
  mutate(group = case_when(group == "Normal" ~ "OAC",
                           group == "Super" ~ "SuperAger"))

# Test for group differences and visualize results
# Install ggpubr
# install.packages('ggpubr')
library(ggpubr)

all_across_networks_summary %>%
  group_by(group) %>%
  # Factor and reorder group (this makes the graphs look nicer)
  mutate(group = factor(group, levels = c('SuperAger', 'OAC')),
         measure = factor(measure, levels = c('Between Network',
                                              'Within Network',
                                              'Segregation'))) %>%
  # Test and plot
  ggplot(aes(y = value, x = group, pattern = group)) +
  facet_wrap(~measure, scales = "free_y") +
  # Run stats
  stat_compare_means(comparisons = list(c("SuperAger", "OAC")),
                     paired = F,
                     method = "wilcox.test",
                     label = "..p.format..") +
  geom_boxplot_pattern(position = "dodge", color = "black", pattern_color = "black", pattern_fill = "black", pattern_angle = 45, pattern_density = 0.1, pattern_spacing = 0.06, pattern_key_scale_factor = 0.1) +
  scale_pattern_manual(values = c(SuperAger = "none", OAC = "stripe")) +
  theme_grey(base_size = 15) +
  guides(
    fill = "none"
  ) +
  labs(
    pattern = "Group",
    y = "Average Functional Connectivity (z)"
  ) +
  theme(
    axis.ticks.x = element_blank(),
    axis.title.x = element_blank(),
    axis.text.x = element_blank()
  ) +
  scale_y_continuous(expand = expansion(add = 0.05))

# Without P-Values

all_across_networks_summary %>%
  group_by(group) %>%
  # Factor and reorder group (this makes the graphs look nicer)
  mutate(group = factor(group, levels = c('SuperAger', 'OAC')),
         measure = factor(measure, levels = c('Between Network',
                                              'Within Network',
                                              'Segregation'))) %>%
  # Test and plot
  ggplot(aes(y = value, x = group, pattern = group)) +
  facet_wrap(~measure, scales = "free_y") +
  geom_boxplot_pattern(position = "dodge", color = "black", pattern_color = "black", pattern_fill = "black", pattern_angle = 45, pattern_density = 0.1, pattern_spacing = 0.06, pattern_key_scale_factor = 0.1) +
  scale_pattern_manual(values = c(SuperAger = "none", OAC = "stripe")) +
  theme_grey(base_size = 15) +
  guides(
    fill = "none"
  ) +
  labs(
    pattern = "Group",
    y = "Average Functional Connectivity (z)"
  ) +
  theme(
    axis.ticks.x = element_blank(),
    axis.title.x = element_blank(),
    axis.text.x = element_blank()
  )

# 7 Network Analysis with GSR -----------

schaefer_407_info <- read.table(file.path(data_release_dir, "Schaefer2018_400Parcels_7Networks_order_info.txt"), sep = "\t", header = F)


# Clean up dataframe
schaefer_407_info <- schaefer_407_info %>%
  # Separate parcel info and color
  mutate(column_name = rep(c("parcel_id_info", "parcel_color"), nrow(schaefer_407_info) / 2),
         key = rep(1:(nrow(schaefer_407_info) / 2), each = 2)) %>%
  pivot_wider(id_cols = key, names_from = column_name, values_from = V1) %>%
  select(-key) %>%
  # Clean up "parcel_id_info" column
  separate_wider_delim(cols = parcel_id_info, delim = "_", names = c("atlas", "hemi", "parc_name"), too_many = "merge") %>%
  # Clean up "parcel_color" column
  separate_wider_delim(cols = parcel_color, delim = " ", names = c("parcel_id", "r", "g", "b", "alpha")) %>%
  # Make network name column
  mutate(net_name = parc_name) %>%
  separate_wider_delim(cols = net_name, delim = "_", names = c("net_name"), too_many = "drop") %>%
  # Make full network name column
  mutate(network_name = case_when(net_name == "Vis" ~ "Visual",
                                  net_name == "SomMot" ~ "Somatomotor",
                                  net_name == "DorsAttn" ~ "Dorsal Attention",
                                  net_name == "SalVentAttn" ~ "Ventral Attention",
                                  net_name == "Limbic" ~ "Limbic",
                                  net_name == "Cont" ~ "Executive Control",
                                  net_name == "Default" ~ "Default Mode")) %>%
  # Replace r b g with hex (and remove alpha); first average r, g, b within network because they are slightly different
  group_by(network_name) %>%
  reframe(m_r = mean(as.numeric(r)),
          m_g = mean(as.numeric(g)),
          m_b = mean(as.numeric(b)),
          across()) %>%
  mutate(rgb = rgb(m_r, m_g, m_b, maxColorValue = 255)) %>%
  select(-r, -g, -b, -m_r, -m_g, -m_b, -alpha) %>%
  # Finally, factor these color; this is helpful for visualization
  mutate(rgb = factor(rgb, levels = c("#791286",
                                      "#4882B4",
                                      "#02760F",
                                      "#C639FB",
                                      "#DEF8A7",
                                      "#E89423",
                                      "#CE3E4F")))

# Create summary dataframe of correlation matrices (pconn)

all_pconn_dataframes_list = list()

for (i in 1:nrow(subject_list)) {

  # Assign sub ID
  sub <- subject_list[i,]

  # Location of pconn
  pconn_file <- file.path(data_release_dir, "Raw_Data/GSR/7network", list.files(path = file.path(data_release_dir, "Raw_Data/GSR/7network"),  pattern = sub))

  # Read in pconn
  pconn <- read.table(pconn_file, sep = "\t", header = F)

  # # Remove the top triangle (and diagonal) of the pconn
  # pconn[upper.tri(as.matrix(pconn), diag = T)] = NA

  # Make column name column number (i.e., parcel_id)
  colnames(pconn) <- c(1:400)

  # Install reshape2 for melt() function
  # install.packages('reshape2')
  library(reshape2)

  # Turn correlation matrix into long dataframe
  pconn_dataframe <- melt(as.matrix(pconn), value.name = "r_value") %>%
    # Remove NA values (i.e. the top triangle and diagonal)
    drop_na()

  # Convert r values to Fisher's z-scores
  # Install correlation package
  # install.packages('correlation')
  library(correlation)

  pconn_dataframe <- pconn_dataframe %>%
    mutate(z_value = z_fisher(r = r_value)) %>%
    select(-r_value)

  # Finally, add subject id
  pconn_dataframe <- pconn_dataframe %>%
    mutate(subject_id = sub)

  # Add pconn dataframe to growing list of all subject pconn dataframes
  all_pconn_dataframes_list[i] <- list(pconn_dataframe)

}

# Bind all_pconn_dataframes; turn it from a list to a dataframe
all_pconn_dataframes <- bind_rows(all_pconn_dataframes_list)

# Within-network analysis


# Summarize dataframe into within-network functional connectivity (i.e., z_value); summarize for each network and for each subject

# First, add network names for each parcel involved

all_pconn_dataframes <- all_pconn_dataframes %>%
  # Make sure Var1 and Var2 are characters (they are technically parcel_id)
  mutate(Var1 = as.character(Var1),
         Var2 = as.character(Var2)) %>%
  # Add columns for networks associated with Var1 and Var2; this is taken from schaefer_407_info defined above
  left_join(., schaefer_407_info %>% select(parcel_id, network_name), by = c("Var1" = "parcel_id")) %>%
  # Change name to reflext network for Var1
  mutate(Var1_network_name = network_name) %>%
  select(-network_name) %>%
  # Do the same for Var2
  left_join(., schaefer_407_info %>% select(parcel_id, network_name), by = c("Var2" = "parcel_id")) %>%
  # Change name to reflext network for Var1
  mutate(Var2_network_name = network_name) %>%
  select(-network_name)

# 7 Network Summary Correlation Matrices -----------

# SuperAgers
all_pconn_dataframes %>%
  left_join(., group_ref) %>%
  # Change names to SuperAger and OAC (older-aged control)
  mutate(group = case_when(group == "Normal" ~ "OAC",
                           group == "Super" ~ "SuperAger")) %>%
  filter(group == "SuperAger") %>%
  group_by(Var1_network_name, Var2_network_name) %>%
  filter(!is.infinite(z_value)) %>%
  summarise(z_value = mean(z_value)) %>%
  ggplot(aes(x = Var1_network_name, y = Var2_network_name, fill = z_value)) +
  geom_raster() +
  theme_bw() +
  # Because we need the x and y axis to display every node,
  # not just the nodes that have connections to each other,
  # make sure that ggplot does not drop unused factor levels
  scale_x_discrete(drop = FALSE) +
  scale_y_discrete(drop = FALSE) +
  theme_grey(base_size = 15) +
  labs(
    title = "SuperAgers"
  ) +
  theme(
    # Rotate the x-axis lables so they are legible
    axis.text.x = element_text(angle=40, vjust=1, hjust=1),
    axis.title = element_blank(),
    plot.title = element_text(hjust = 0.5),
    # Force the plot into a square aspect ratio
    aspect.ratio = 1) +
  scale_fill_gradient2(limits = c(-0.05, 0.35), midpoint = 0, low = "blue", high = "red") +
  labs(
    fill = "Z Value"
  )

# OAC
all_pconn_dataframes %>%
  left_join(., group_ref) %>%
  # Change names to SuperAger and OAC (older-aged control)
  mutate(group = case_when(group == "Normal" ~ "Older-Aged-Control",
                           group == "Super" ~ "SuperAger")) %>%
  filter(group == "Older-Aged-Control") %>%
  group_by(Var1_network_name, Var2_network_name) %>%
  filter(!is.infinite(z_value)) %>%
  summarise(z_value = mean(z_value)) %>%
  ggplot(aes(x = Var1_network_name, y = Var2_network_name, fill = z_value)) +
  geom_raster() +
  theme_bw() +
  # Because we need the x and y axis to display every node,
  # not just the nodes that have connections to each other,
  # make sure that ggplot does not drop unused factor levels
  scale_x_discrete(drop = FALSE) +
  scale_y_discrete(drop = FALSE) +
  theme_grey(base_size = 15) +
  labs(
    title = "Older-Aged-Controls"
  ) +
  theme(
    # Rotate the x-axis lables so they are legible
    axis.text.x = element_text(angle=40, vjust=1, hjust=1),
    axis.title = element_blank(),
    plot.title = element_text(hjust = 0.5),
    # Force the plot into a square aspect ratio
    aspect.ratio = 1) +
  scale_fill_gradient2(limits = c(-0.05, 0.35), midpoint = 0, low = "blue", high = "red") +
  labs(
    fill = "Z Value"
  )

# 7 Network Group Difference Correlation Matrices -----------

all_pconn_dataframes %>%
  left_join(., group_ref) %>%
  # Change names to SuperAger and OAC (older-aged control)
  mutate(group = case_when(group == "Normal" ~ "OAC",
                           group == "Super" ~ "SuperAger")) %>%
  group_by(group, Var1_network_name, Var2_network_name) %>%
  filter(!is.infinite(z_value)) %>%
  summarise(z_value = mean(z_value)) %>%
  pivot_wider(names_from = group, values_from = z_value) %>%
  mutate(diff = SuperAger - OAC) %>%
  ggplot(aes(x = Var1_network_name, y = Var2_network_name, fill = diff)) +
  geom_raster() +
  theme_bw() +
  # Because we need the x and y axis to display every node,
  # not just the nodes that have connections to each other,
  # make sure that ggplot does not drop unused factor levels
  scale_x_discrete(drop = FALSE) +
  scale_y_discrete(drop = FALSE) +
  theme_grey(base_size = 15) +
  labs(
    title = "SuperAger - Older-Aged-Control"
  ) +
  theme(
    # Rotate the x-axis lables so they are legible
    axis.text.x = element_text(angle=40, vjust=1, hjust=1),
    axis.title = element_blank(),
    plot.title = element_text(hjust = 0.5),
    # Force the plot into a square aspect ratio
    aspect.ratio = 1) +
  scale_fill_gradient2(midpoint = 0, low = "blue", high = "red") +
  labs(
    fill = "Z Value"
  )

# 7 Network Within Networks -----------

all_pconn_dataframes %>%
  filter(!is.infinite(z_value),
         Var1_network_name == Var2_network_name) %>%
  group_by(subject_id, Var1_network_name) %>%
  summarise(avg_z_value = mean(z_value)) %>%
  ungroup() %>%
  left_join(., group_ref) %>%
  # Change names to SuperAger and OAC (older-aged control)
  mutate(group = case_when(group == "Normal" ~ "OAC",
                           group == "Super" ~ "SuperAger")) %>%
  rename(network_name = Var1_network_name) %>%
  # Factor and reorder group and networks (this makes the graphs look nicer)
  mutate(group = factor(group, levels = c('SuperAger', 'OAC')),
         network_name = factor(network_name, levels = c("Visual",
                                                        "Somatomotor",
                                                        "Dorsal Attention",
                                                        "Ventral Attention",
                                                        "Limbic",
                                                        "Executive Control",
                                                        "Default Mode"))) %>%
  # Test and plot
  ggplot(aes(y = avg_z_value, x = group, pattern = group)) +
  facet_wrap(~network_name, scales = "free_y", nrow = 2) +
  # Run stats
  stat_compare_means(comparisons = list(c("SuperAger", "OAC")),
                     paired = F,
                     method = "wilcox.test",
                     label = "..p.format..") +
  geom_boxplot_pattern(position = "dodge", aes(fill = network_name), color = "black", pattern_color = "black", pattern_fill = "black", pattern_angle = 45, pattern_density = 0.1, pattern_spacing = 0.06, pattern_key_scale_factor = 0.1) +
  # Get colors from schaefer_407_info
  theme_grey(base_size = 15) +
  scale_fill_manual(values = c(as.character(sort(unique(schaefer_407_info$rgb))))) +
  scale_pattern_manual(values = c(SuperAger = "none", OAC = "stripe"),) +
  labs(
    fill = "Resting State Network",
    y = "Average Functional Connectivity (z)",
    pattern = "Group"
  ) +
  theme(
    axis.ticks.x = element_blank(),
    axis.title.x = element_blank(),
    axis.text.x = element_blank()
  ) +
  scale_y_continuous(expand = expansion(add = 0.05))

# Without P-Values

all_pconn_dataframes %>%
  filter(!is.infinite(z_value),
         Var1_network_name == Var2_network_name) %>%
  group_by(subject_id, Var1_network_name) %>%
  summarise(avg_z_value = mean(z_value)) %>%
  ungroup() %>%
  left_join(., group_ref) %>%
  # Change names to SuperAger and OAC (older-aged control)
  mutate(group = case_when(group == "Normal" ~ "OAC",
                           group == "Super" ~ "SuperAger")) %>%
  rename(network_name = Var1_network_name) %>%
  # Factor and reorder group and networks (this makes the graphs look nicer)
  mutate(group = factor(group, levels = c('SuperAger', 'OAC')),
         network_name = factor(network_name, levels = c("Visual",
                                                        "Somatomotor",
                                                        "Dorsal Attention",
                                                        "Ventral Attention",
                                                        "Limbic",
                                                        "Executive Control",
                                                        "Default Mode"))) %>%
  # Test and plot
  ggplot(aes(y = avg_z_value, x = group, pattern = group)) +
  facet_wrap(~network_name, scales = "free_y", nrow = 2) +
  geom_boxplot_pattern(position = "dodge", aes(fill = network_name), color = "black", pattern_color = "black", pattern_fill = "black", pattern_angle = 45, pattern_density = 0.1, pattern_spacing = 0.06, pattern_key_scale_factor = 0.1) +
  # Get colors from schaefer_407_info
  theme_grey(base_size = 15) +
  scale_fill_manual(values = c(as.character(sort(unique(schaefer_407_info$rgb))))) +
  scale_pattern_manual(values = c(SuperAger = "none", OAC = "stripe"),) +
  labs(
    fill = "Resting State Network",
    y = "Average Functional Connectivity (z)",
    pattern = "Group"
  ) +
  theme(
    axis.ticks.x = element_blank(),
    axis.title.x = element_blank(),
    axis.text.x = element_blank()
  )

# For graphical abstract

all_pconn_dataframes %>%
  filter(!is.infinite(z_value),
         Var1_network_name == Var2_network_name) %>%
  group_by(subject_id, Var1_network_name) %>%
  summarise(avg_z_value = mean(z_value)) %>%
  ungroup() %>%
  left_join(., group_ref) %>%
  # Change names to SuperAger and OAC (older-aged control)
  mutate(group = case_when(group == "Normal" ~ "OAC",
                           group == "Super" ~ "SuperAger")) %>%
  rename(network_name = Var1_network_name) %>%
  # Factor and reorder group and networks (this makes the graphs look nicer)
  mutate(group = factor(group, levels = c('SuperAger', 'OAC')),
         network_name = factor(network_name, levels = c("Visual",
                                                        "Somatomotor",
                                                        "Dorsal Attention",
                                                        "Ventral Attention",
                                                        "Limbic",
                                                        "Executive Control",
                                                        "Default Mode"))) %>%
  # Test and plot
  ggplot(aes(y = avg_z_value, x = group, pattern = group)) +
  facet_wrap(~network_name, scales = "free_y", nrow = 2) +
  geom_boxplot_pattern(position = "dodge", aes(fill = network_name), color = "black", pattern_color = "black", pattern_fill = "black", pattern_angle = 45, pattern_density = 0.1, pattern_spacing = 0.06, pattern_key_scale_factor = 0.1) +
  # Get colors from schaefer_407_info
  theme_grey(base_size = 15) +
  theme_classic() +
  scale_fill_manual(values = c(as.character(sort(unique(schaefer_407_info$rgb))))) +
  scale_pattern_manual(values = c(SuperAger = "none", OAC = "stripe"),) +
  labs(
    fill = "Resting State Network",
    y = "Average Functional Connectivity (z)",
    pattern = "Group"
  ) +
  theme(
    axis.ticks.x = element_blank(),
    axis.title.x = element_blank(),
    axis.text.x = element_blank()
  )

# 7 Network Between Networks -----------

all_pconn_dataframes %>%
  filter(!is.infinite(z_value),
         Var1_network_name != Var2_network_name) %>%
  group_by(subject_id, Var1_network_name, Var2_network_name) %>%
  summarise(avg_z_value = mean(z_value)) %>%
  ungroup() %>%
  left_join(., group_ref) %>%
  # Change names to SuperAger and OAC (older-aged control)
  mutate(group = case_when(group == "Normal" ~ "OAC",
                           group == "Super" ~ "SuperAger")) %>%
  # Factor and reorder group and networks (this makes the graphs look nicer)
  mutate(group = factor(group, levels = c('SuperAger', 'OAC')),
         Var1_network_name = factor(Var1_network_name, levels = c("Visual",
                                                                  "Somatomotor",
                                                                  "Dorsal Attention",
                                                                  "Ventral Attention",
                                                                  "Limbic",
                                                                  "Executive Control",
                                                                  "Default Mode")),
         Var2_network_name = factor(Var2_network_name, levels = c("Visual",
                                                                  "Somatomotor",
                                                                  "Dorsal Attention",
                                                                  "Ventral Attention",
                                                                  "Limbic",
                                                                  "Executive Control",
                                                                  "Default Mode"))) %>%
  # Test and plot
  ggplot(aes(y = avg_z_value, x = Var2_network_name, pattern = group)) +
  facet_wrap(~Var1_network_name, scales = "free", nrow = 2) +
  # Run stats
  stat_compare_means(aes(group = group),
                     paired = F,
                     method = "wilcox.test",
                     label = "..p.format..") +
  geom_boxplot_pattern(position = "dodge", aes(fill = Var1_network_name), color = "black", pattern_color = "black", pattern_fill = "black", pattern_angle = 45, pattern_density = 0.1, pattern_spacing = 0.06, pattern_key_scale_factor = 0.1) +
  # Get colors from schaefer_407_info
  theme_grey(base_size = 15) +
  scale_fill_manual(values = c(as.character(sort(unique(schaefer_407_info$rgb))))) +
  scale_pattern_manual(values = c(SuperAger = "none", OAC = "stripe"),) +
  labs(
    fill = "Resting State Network",
    y = "Average Functional Connectivity (z)",
    pattern = "Group"
  ) +
  theme(
    axis.ticks.x = element_blank(),
    axis.title.x = element_blank(),
    axis.text.x = element_text(angle=40, vjust=1, hjust=1)
  ) +
  scale_y_continuous(expand = expansion(add = 0.1))

# No P-Values

all_pconn_dataframes %>%
  filter(!is.infinite(z_value),
         Var1_network_name != Var2_network_name) %>%
  group_by(subject_id, Var1_network_name, Var2_network_name) %>%
  summarise(avg_z_value = mean(z_value)) %>%
  ungroup() %>%
  left_join(., group_ref) %>%
  # Change names to SuperAger and OAC (older-aged control)
  mutate(group = case_when(group == "Normal" ~ "OAC",
                           group == "Super" ~ "SuperAger")) %>%
  # Factor and reorder group and networks (this makes the graphs look nicer)
  mutate(group = factor(group, levels = c('SuperAger', 'OAC')),
         Var1_network_name = factor(Var1_network_name, levels = c("Visual",
                                                                  "Somatomotor",
                                                                  "Dorsal Attention",
                                                                  "Ventral Attention",
                                                                  "Limbic",
                                                                  "Executive Control",
                                                                  "Default Mode")),
         Var2_network_name = factor(Var2_network_name, levels = c("Visual",
                                                                  "Somatomotor",
                                                                  "Dorsal Attention",
                                                                  "Ventral Attention",
                                                                  "Limbic",
                                                                  "Executive Control",
                                                                  "Default Mode"))) %>%
  # Test and plot
  ggplot(aes(y = avg_z_value, x = Var2_network_name, pattern = group)) +
  facet_wrap(~Var1_network_name, scales = "free", nrow = 2) +
  geom_boxplot_pattern(position = "dodge", aes(fill = Var1_network_name), color = "black", pattern_color = "black", pattern_fill = "black", pattern_angle = 45, pattern_density = 0.1, pattern_spacing = 0.06, pattern_key_scale_factor = 0.1) +
  # Get colors from schaefer_407_info
  theme_grey(base_size = 15) +
  scale_fill_manual(values = c(as.character(sort(unique(schaefer_407_info$rgb))))) +
  scale_pattern_manual(values = c(SuperAger = "none", OAC = "stripe"),) +
  labs(
    fill = "Resting State Network",
    y = "Average Functional Connectivity (z)",
    pattern = "Group"
  ) +
  theme(
    axis.ticks.x = element_blank(),
    axis.title.x = element_blank(),
    axis.text.x = element_text(angle=40, vjust=1, hjust=1)
  )

# For graphical abstract

all_pconn_dataframes %>%
  filter(!is.infinite(z_value),
         Var1_network_name != Var2_network_name) %>%
  group_by(subject_id, Var1_network_name, Var2_network_name) %>%
  summarise(avg_z_value = mean(z_value)) %>%
  ungroup() %>%
  left_join(., group_ref) %>%
  # Change names to SuperAger and OAC (older-aged control)
  mutate(group = case_when(group == "Normal" ~ "OAC",
                           group == "Super" ~ "SuperAger")) %>%
  # Factor and reorder group and networks (this makes the graphs look nicer)
  mutate(group = factor(group, levels = c('SuperAger', 'OAC')),
         Var1_network_name = factor(Var1_network_name, levels = c("Visual",
                                                                  "Somatomotor",
                                                                  "Dorsal Attention",
                                                                  "Ventral Attention",
                                                                  "Limbic",
                                                                  "Executive Control",
                                                                  "Default Mode")),
         Var2_network_name = factor(Var2_network_name, levels = c("Visual",
                                                                  "Somatomotor",
                                                                  "Dorsal Attention",
                                                                  "Ventral Attention",
                                                                  "Limbic",
                                                                  "Executive Control",
                                                                  "Default Mode"))) %>%
  # Test and plot
  ggplot(aes(y = avg_z_value, x = Var2_network_name, pattern = group)) +
  facet_wrap(~Var1_network_name, scales = "free", nrow = 2) +
  geom_boxplot_pattern(position = "dodge", aes(fill = Var1_network_name), color = "black", pattern_color = "black", pattern_fill = "black", pattern_angle = 45, pattern_density = 0.1, pattern_spacing = 0.06, pattern_key_scale_factor = 0.1) +
  # Get colors from schaefer_407_info
  theme_grey(base_size = 15) +
  theme_classic2()+
  scale_fill_manual(values = c(as.character(sort(unique(schaefer_407_info$rgb))))) +
  scale_pattern_manual(values = c(SuperAger = "none", OAC = "stripe"),) +
  labs(
    fill = "Resting State Network",
    y = "Average Functional Connectivity (z)",
    pattern = "Group"
  ) +
  theme(
    axis.ticks.x = element_blank(),
    axis.title.x = element_blank(),
    axis.text.x = element_text(angle=40, vjust=1, hjust=1)
  )

# 7 Network Average Within/Between/Segregation -----------

# Summarize dataframe into between-network functional connectivity (i.e., z_value); summarize for each subject

# Second, summarize across networks within-network and between-network averages for each subject

all_across_networks_summary <- all_pconn_dataframes %>%
  filter(!is.infinite(z_value)) %>%
  # Add column to check if networks are the same
  mutate(within_network = ifelse(Var1_network_name == Var2_network_name, "Within Network", "Between Network")) %>%
  # Average within subjects
  group_by(subject_id, within_network) %>%
  summarise(z_value = mean(z_value)) %>%
  ungroup() %>%
  # Calculate segregation value
  pivot_wider(names_from = within_network, values_from = z_value) %>%
  mutate(Segregation = (`Within Network` - `Between Network`) / `Within Network`) %>%
  pivot_longer(cols = c(`Within Network`, `Between Network`, Segregation),
               names_to = "measure",
               values_to = "value") %>%
  left_join(., group_ref, by = join_by(subject_id)) %>%
  # Change names to SuperAger and OAC (older-aged control)
  mutate(group = case_when(group == "Normal" ~ "OAC",
                           group == "Super" ~ "SuperAger"))

# Test for group differences and visualize results
# Install ggpubr
# install.packages('ggpubr')
library(ggpubr)

all_across_networks_summary %>%
  group_by(group) %>%
  # Factor and reorder group (this makes the graphs look nicer)
  mutate(group = factor(group, levels = c('SuperAger', 'OAC')),
         measure = factor(measure, levels = c('Between Network',
                                              'Within Network',
                                              'Segregation'))) %>%
  # Test and plot
  ggplot(aes(y = value, x = group, pattern = group)) +
  facet_wrap(~measure, scales = "free_y") +
  # Run stats
  stat_compare_means(comparisons = list(c("SuperAger", "OAC")),
                     paired = F,
                     method = "wilcox.test",
                     label = "..p.format..") +
  geom_boxplot_pattern(position = "dodge", color = "black", pattern_color = "black", pattern_fill = "black", pattern_angle = 45, pattern_density = 0.1, pattern_spacing = 0.06, pattern_key_scale_factor = 0.1) +
  scale_pattern_manual(values = c(SuperAger = "none", OAC = "stripe")) +
  theme_grey(base_size = 15) +
  guides(
    fill = "none"
  ) +
  labs(
    pattern = "Group",
    y = "Average Functional Connectivity (z)"
  ) +
  theme(
    axis.ticks.x = element_blank(),
    axis.title.x = element_blank(),
    axis.text.x = element_blank()
  ) +
  scale_y_continuous(expand = expansion(add = 0.05))

# Without P-Values

all_across_networks_summary %>%
  group_by(group) %>%
  # Factor and reorder group (this makes the graphs look nicer)
  mutate(group = factor(group, levels = c('SuperAger', 'OAC')),
         measure = factor(measure, levels = c('Between Network',
                                              'Within Network',
                                              'Segregation'))) %>%
  # Test and plot
  ggplot(aes(y = value, x = group, pattern = group)) +
  facet_wrap(~measure, scales = "free_y") +
  geom_boxplot_pattern(position = "dodge", color = "black", pattern_color = "black", pattern_fill = "black", pattern_angle = 45, pattern_density = 0.1, pattern_spacing = 0.06, pattern_key_scale_factor = 0.1) +
  scale_pattern_manual(values = c(SuperAger = "none", OAC = "stripe")) +
  theme_grey(base_size = 15) +
  guides(
    fill = "none"
  ) +
  labs(
    pattern = "Group",
    y = "Average Functional Connectivity (z)"
  ) +
  theme(
    axis.ticks.x = element_blank(),
    axis.title.x = element_blank(),
    axis.text.x = element_blank()
  )
```
